# Prioritization of the Skills to Be Mastered for the Daily Jobs of Japanese Dental Hygienists

**DOI:** 10.1155/2020/4297646

**Published:** 2020-06-22

**Authors:** Yoshiaki Nomura, Erika Kakuta, Ayako Okada, Yuko Yamamoto, Hiroshi Tomonari, Noriyasu Hosoya, Nobuhiro Hanada, Naomi Yoshida, Noriko Takei

**Affiliations:** ^1^Department of Translational Research, Tsurumi University School of Dental Medicine, Yokohama, Japan; ^2^Department of Oral Microbiology, Tsurumi University School of Dental Medicine, Yokohama, Japan; ^3^Department of Operative Dentistry, Tsurumi University School of Dental Medicine, Yokohama, Japan; ^4^Department of Orthodontics, Tsurumi University School of Dental Medicine, Yokohama, Japan; ^5^Department of Endodontics, Tsurumi University School of Dental Medicine, Yokohama, Japan; ^6^Japanese Dental Hygienists' Association, Tokyo, Japan

## Abstract

Dental hygienists require proficiency in a wide variety of job skills. Dental hygienists should master their job skills step by step, and the prioritization of these steps is important. In this study, we investigated the frequency at which Japanese dental hygienists performed daily jobs and attempted to classify the jobs according to the proficiency levels. The aim of this study was to surmise the order in which skills should be mastered in terms of priority and to investigate the relationship between daily jobs and the motivation for completing jobs. The Japan Dental Hygienists' Association conducts a survey on the employment status of dental hygienists in Japan every five years. The questionnaire is distributed to all members of the Japan Dental Hygienists' Association. In this study, the responses of 3,807 dental hygienists who worked at dental clinics were analyzed. We analyzed 77 kinds of daily jobs and the items regarding the motivation to work. For the analysis, item response theory (IRT), structural equation modeling (SEM), and logistic regression analysis were applied. According to the item response curve, the jobs were classified into 11 clusters. The jobs classified into Cluster 1 were the jobs that most of the average-proficiency Japanese dental hygienists performed. Scaling and root planing were the representative jobs in Cluster 1. Performing the jobs classified into Cluster 5 clearly discriminated whether the dental hygienists were performing multiple jobs. Jobs concerning care for elderly or disabled patients were classified into Cluster 5. Jobs concerning gerodontology, implants, management of staff, and consultations were significantly associated with the motivation to perform jobs. Polishing and adjustment of orthodontic apparatuses was negatively associated with the motivation to perform jobs. Understanding the features of each daily job of dental hygienists is important for planning dental hygienists' lifelong educational programs and evaluating their skill levels and proficiency levels. The results presented in this study may help to reveal the characteristics of dental hygienists' daily jobs.

## 1. Introduction

Dental hygienists perform important work to promote oral health [1–3]. Dental hygienists require a national license to practice in Japan. The Japanese legislation defines a dental hygienist as a health care professional engaged in the prevention of dental and oral diseases. The work of a dental hygienist includes mechanically removing deposits from tooth surfaces and from the location below the free gingival margin, administering fluoride varnish on tooth surfaces, and assisting in providing dental care and instructions for oral hygiene. However, in practice, there are a wide variety of job descriptions.

Traditionally, the education required for dental hygienists in Japan has included a two-year program, but the Japanese government changed the educational and legal system for dental hygienists. Since 2004, several universities have started offering four-year educational programs for dental hygienists, and some of them have opened master's degree courses. Since 2005, a three-year educational program has been required in the program offerings of all Japanese dental hygienist schools. The Ministry of Education, Culture, Sports, Science and Technology of Japan proposed the minimum educational requirements to be included in the core curriculum. However, the curriculum of the advanced educational program is not standardized. The curriculum of the advanced educational program varies between schools. When planning an advanced educational program, we need to consider the frequency of daily jobs and the proficiency levels required for mastering skills. This information is also useful for lifelong educational programs.

Japan is now a super-aged society. There are many elderly people with disabilities in nursing homes or in their own homes. Beyond providing oral health care, dental hygienists and dentists are expected to contribute to overall health care and quality of life, and they are expected to highlight and integrate the correlation between oral and systemic diseases [4, 5]. With changes in social demands and advances in dentistry, dental hygienists must acquire extensive knowledge and skills. In addition to the conventional knowledge and skills in oral health care techniques, health care and nursing skills are essential to promote health, especially for elderly or disabled patients.

The educational program must be in line with the actual jobs that dental hygienists perform daily. However, mastering all of the required skills for a dental hygienist within the 3 years allotted for graduate programs is impossible. Thus, an advanced technical educational program is necessary. For the planning of an advanced educational program, the daily frequency of the job and the priority in which skills should be mastered are important to understand. There are reports that show the daily frequency of dental hygienists' jobs [6, 7]. However, these reports present only descriptive analyses of the dental hygienists' jobs.

In addition to improving the proficiency and technical skills of dental hygienists, improving the job and carrier satisfaction of dental hygienists is important [8–11]. Job satisfaction contributes positively to the well-being of workers [12]. Satisfaction is assumed to be related to both job and career attrition [13, 14]. Skill variety is one of the key factors for dental hygienist job satisfaction [15]. Dental hygienists should master their job skills step by step, and the order in which the skills are mastered is important for acquiring these skills. The frequency, difficulty, and required proficiency levels of these skills are important determinants of the order in which the skills should be mastered.

The aim of this study was to investigate the frequency and proficiency levels of daily jobs of Japanese dental hygienists. This information may be useful in planning lifelong educational programs for dental hygienists. Additionally, their contributions to job satisfaction may be useful information for improving educational programs.

## 2. Methods

### 2.1. Survey Method

The Japan Dental Hygienists' Association has conducted a survey on the employment status of dental hygienists in Japan every five years since 1981. Because this survey was supported by the Japanese government, it conformed to the national survey. On September 30, 2014, the questionnaire, including a stamped envelope for return, was distributed to all members of the Japan Dental Hygienists' Association by mail. The survey date was set for October 31. The questionnaires returned through November 30 were used for analysis.

### 2.2. Questionnaire

The questionnaire used in this study consisted 94 major items related to demographic factors, employment status, work content, and willingness to work. We analyzed 77 kinds of daily work-related tasks of dental hygienists.

For the items regarding the motivation of dental hygienists, two items were used: “the work as a dental hygienist is worthwhile” and “willingness to continue working as a dental hygienist.”

### 2.3. Statistical Analysis

A three-parameter logistic model with item response theory (IRT) analysis was applied to calculate the item discriminations, item difficulties, and item guesses for the work-related tasks. Item response curves and item information curves were graphically illustrated. The analyses were carried out by the *R* software with the LTR and irtoys packages using the following formula:

(1) $$\begin{array}{c} \left(\left.U_{i,j}=1\right|\theta_{i},a_{j},b_{j,}C_{j}\right)=C_{j}+\frac{\left(1-c_{j}\right)}{1+\exp\left(a_{j}\left(\theta_{i}-b_{j}\right)\right)}. \end{array}$$

Factor analysis with varimax rotation was carried out to determine the latent variables for structural equation modeling (SEM). The structural relationship between the work-related tasks and motivation of dental hygienists was calculated by the AMOS software (AMOS ver. 24.0, IBM, Tokyo, Japan).

Odds ratios for the motivation to work were calculated by logistic regression analysis using the work-related tasks as independent variables. The responses to the “work of a dental hygienist is worthwhile” item were recorded on a four-point Likert scale: “strongly agree,” “agree,” “disagree,” and “strongly disagree.” For the logistic regression analysis, the data were dichotomized as “agree” and “disagree.” For the analysis, all seventy seven items about work-related tasks were used, and statistically significant items were selected by the stepwise method. SPSS Statistics Ver. 24.0 (IBM, Tokyo) was used for analyses other than the IRT and SEM analyses.

### 2.4. Ethical Approval and Consent to Participate

This study was approved by the Ethics Committee of the Tsurumi University School of Dental Medicine (approval number: 1633) and conducted in accordance with the Declaration of Helsinki. Informed written consent was obtained from all participants.

## 3. Results

The study population included all members of the Japan Dental Hygienists' Association. The questionnaire was distributed to all 16,113 members of the association. A total of 8,780 responses were recovered, and the collection rate was 54.5%. The study population included civil servants, hospital workers, and retired dental hygienists. Of these individuals, the data of 3,807 dental hygienists who work in private dental clinics were analyzed in this study. There were 2,133 full-time employees (56.9%) and 1,618 part-time employees (43.1%). The average period for which these individuals worked as a dental hygienist was 16.36 ± 10.37 years.

First, by using the item response theory (IRT), we analyzed 77 specific jobs and whether they were performed by each respondent. A three-parameter logistic model was applied to describe the item response curves and item information curves. The frequency at which the specific seventy seven jobs were performed and the parameter estimates of the three-parameter logistic model are shown in Table S1. All item response curves and item information curves are shown in Figure S1. For the item response curve, the intercept of the item response curve indicated the percentage of jobs performed by average-proficiency Japanese dental hygienists. The probability of a correct answer probability against Ability 4 indicated the percentage of specific jobs performed by Japanese dental hygienists who perform various types of dental hygienist jobs. The probability of a correct answer probability against Ability −4 refers to the proportion of jobs performed by Japanese dental hygienists who perform a minimal amount of dental hygienist jobs. The seventy seven items were classified into 11 clusters according to the correct response rates and item response curve types.

The results of clustering are as follows:

  Cluster 1: probability against Ability 4 was 1, and the intercept was more than 0.9
  Cluster 2: probability against Ability 4 was 1, and the intercept was less than 0.9 and more than 0.5
  Cluster 3: probability against Ability 4 was 1, and the intercept was less than 0.5 and more than 0.25
  Cluster 4: probability against Ability 4 was 1, and the intercept was less than 0.25
  Cluster 5: the criteria for Clusters 1 to 4 were met, and the item response curves showed a perpendicular slope
  Cluster 6: probability against Ability 4 was less than 1 and more than 0.9, and the intercept was more than 0.20
  Cluster 7: probability against Ability 4 was less than 1 and more than 0.9, and the intercept was less than 0.20
  Cluster 8: probability against Ability 4 was less than 0.9 and more than 0.8
  Cluster 9: probability against Ability 4 was less than 0.8
  Cluster 10: the criteria for Clusters 1 and 2 were met, and probability against Ability 0 was between 0 and 0.15
  Cluster 11: the item response curve showed a relatively flat slope

**Figure 1:**
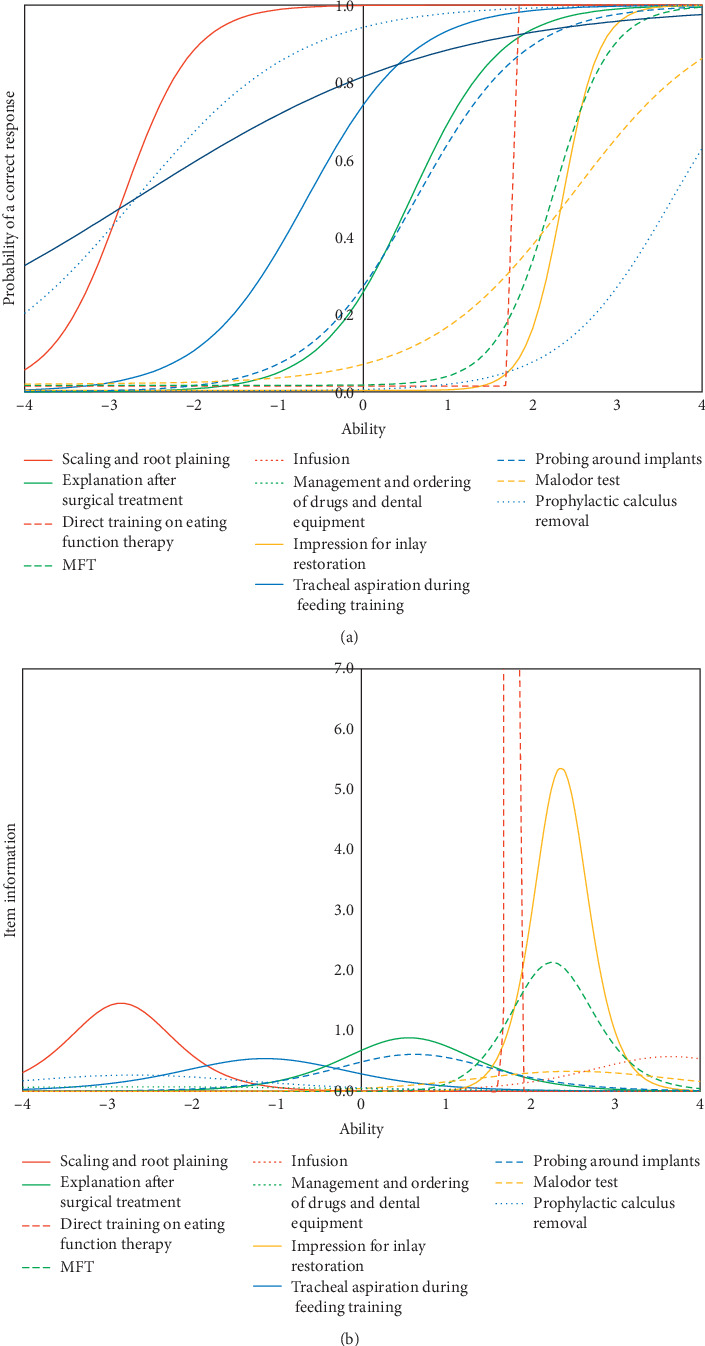
The item response curves and item information curves of the representative items from each cluster. The horizontal axis, known as ability according to item response theory, indicates the dental hygienists who performed a number of daily dental jobs. The vertical axis of the item response curve indicates the percentage of individuals who perform each work-related task. The vertical axis of the item information curve indicated the ability of each item to distinguish the number of daily dental hygienist jobs. Details of the clusters are shown in Table S1.

The item response curves and item information curves of representative items from the 11 clusters are shown in Figure 1. The items with the highest amount of information in each cluster were selected as the representative items.

The item response curves from Cluster 1 corresponding to scaling and root planing showed that most of the average-proficiency Japanese dental hygienists performed this job. Therefore, the peak of the item information curve was shifted to the left side. In contrast to the items for scaling and root planing, the item from Cluster 10 that corresponds to prophylactic calculus removal and that from Cluster 11 that corresponds to the management and ordering of drugs and dental equipment showed high probabilities of a correct response probability against Ability −4. The results showed that these jobs were performed by dental hygienists who were performing a minimal number of jobs. The item response curve from Cluster 2 corresponding to impressions for occlusal splints was shifted more to the right side than was that corresponding to scaling and root planing, and the intercept of it was 0.74. This result showed that after performing the jobs classified into Cluster 1, a relatively high proportion of the average-proficiency Japanese dental hygienists performed the jobs classified into Cluster 2. The intercept of the curve from Cluster 6 corresponding to probing around the implant and that of the curve from Cluster 3 corresponding to the explanation after surgical treatment were less than 0.3, which indicated that fewer than thirty percent of the average-proficiency dental hygienists performed these jobs. The difference between these clusters was whether the correct response probability against Ability 4 was 1.

The item response curve from Cluster 4 corresponding to training on tracheal aspiration during feeding and that from Cluster 7 corresponding to MFT was shifted to the right side. The intercept of these items was very low. Most of the average-proficiency Japanese dental hygienists did not perform these jobs.

The item response curve from Cluster 5 corresponding to direct training on eating function therapy showed a vertical gradient and was extremely sharp. The amount of information on this item was extremely high, and the peak of it was shifted to the right. Whether performing these jobs discriminated the dental hygienists who performed multiple jobs was investigated. The probability of a correct response probability against Ability 4 for the item from Cluster 8 corresponding to the oral malodor test and that from Cluster 9 corresponding to infusion was less than 0.9. This finding indicated that these jobs were not performed by all of the dental hygienists who performed multiple jobs. Few dental hygienists, particularly specialized Japanese dental hygienists, performed the jobs clustered into Clusters 8 and 9.

According to IRT, one's ability, which is a magnitude of a latent trait of an individual, can be calculated. Ability is the capacity or attribute measured by the questionnaire. The mean values of the ability corresponding to each cluster with respect to the age groups were calculated. Among the ability levels corresponding to the eleven clusters, those corresponding to eight clusters were statistically significantly different across the age groups (Figure S2). For Clusters 1, 2, 3, and 10, the abilities decreased with increasing age. These results indicated that these jobs were performed by younger dental hygienists. In contrast, the abilities corresponding to Clusters 4 and 5 were increased in experienced dental hygienists.

Similarly, the mean values of the ability corresponding to each cluster with respect to the workers' employment status (full time or part time) were calculated. The abilities of the full-time workers were higher than those of the part-time workers, except for those corresponding to Cluster 3 (Figure S3). The cross tabulations of each job with respect to the age groups and employment status are shown in Table S2.

After analyzing the characteristics of the seventy seven items, we analyzed the contributions of the daily jobs to job motivation. Regarding the motivation for a job, two items in the questionnaire, the “willingness to work as a dental hygienist” and “willingness to continue to work as a dental hygienist” items, were used as objective variables.

**Figure 2:**
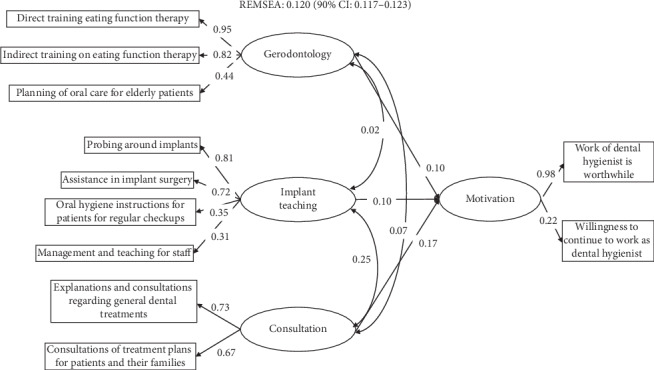
Path diagram of the motivation of dental hygienists based on the work-related tasks. All paths were statistically significant. The fitness index, which was calculated as REMSEA, was 0.120.

First, structural equation modeling was performed to assess the construct validity. Factor analysis was performed before structural equation modeling to construct the latent variables. The results are shown in Table S3. Three factors were extracted, and we named these three factors as jobs concerning “gerodontology,” “implants and teaching,” and “consultations.” Based on the results of the factor analysis, a path diagram was constructed. The results of structural equation modeling are shown in Figure 2. The paths from “gerodontology” and “implants and management of staff members” to “motivation” were 0.10. The path from “consultations” to “motivation” was 0.17. Although all paths were statistically significant, the coefficients were low.

Then, to determine the specific jobs that affect motivation, logistic regression analysis and correspondence analysis were carried out. The results of the logistic regression analysis are shown in Table 1. Items were selected by the stepwise method. For the “willingness to work” item, odds ratios corresponding to “indirect training of eating function therapy” and “planning of oral care programs for elderly patients” were greater than 2.0. The items concerning instructions for the patients, “consultations for treatment plans for patients and their families,” and “explanations and consultations regarding general dental treatment” were included, as were the items concerning the management of human resources, “management and teaching for staff,” and “training and management of dental hygienist students.” The odds ratio of “management and ordering of drugs and dental equipment” was less than 1. This result indicates that this job reduces the motivation to work. For the “willingness to continue to work as a dental hygienist,” jobs concerning implant therapy and gerodontology were included. SPT was included for both “willingness to work” and “willingness to continue to work as a dental hygienist.” The odds ratio of the “polishing and adjustment of orthodontic apparatuses” was less than 1. This finding indicated that this work reduced job motivation.

## 4. Discussion

Planning educational programs for dental hygienists should be based on social demands. Mastering required skills is a step-by-step process that should be performed according to proficiency. There are reports concerning the daily work-related tasks of dental hygienists [6, 7]. These reports presented only descriptive statistics. Descriptive statistics are important for determining facts; however, it is impossible to plan educational programs or improve working conditions by using only descriptive statistics. Statistical inferences made by using only descriptive statistics can be misleading. Confounders or other statistical problems cannot be determined only by descriptive statistics. For this purpose, statistical modeling is indispensable. In this study, we applied item response theory (IRT), structural equation modeling (SEM), and logistic regression analysis to infer the required proficiency levels of the daily jobs of Japanese dental hygienists and the relationship between their daily jobs and motivation to work.

The benefits of IRT include comprehensive analyses, a reduction in measurement errors, the ability to create tests or questionnaires, meaningful scaling of latent variables, objective calibrations and equating, evaluations of tests or questionnaires and item biases, high accuracy in assessments, and evaluations of models and person-fit models [16]. IRT refers to a family of mathematical models that attempt to explain the relationships between latent traits and their manifestations. With IRT, a link is established between the properties of items on an instrument, individuals responding to these items, and the underlying traits being measured. IRT assumes that the latent construct and items of a measure are organized in an unobservable continuum. The underlying traits are usually represented by the total weighted sums of the responses to the items. Using the traditional sum score generally results in high type 1 error rates or low statistical power for detecting curvilinearity, depending on the distribution of the item locations [17]. IRT can establish an individual's position on the continuum.

The results from IRT analysis provided us with valuable information. The proficiency levels with respect to the frequency at which daily jobs were performed could be classified. This result may be related to the skill levels of dental hygienists according to their experiences.

According to the classification constructed by IRT, Cluster 1 had low item difficulty. Daily jobs concerning periodontal treatments and oral hygiene instructions were included in Cluster 1. The daily jobs classified into Cluster 1 need to be taught to dental hygienist novices.

The jobs concerning prosthetic treatments were classified into Cluster 2. The difficulty of the jobs in Cluster 2 was negative. This result showed that these items may be daily jobs for dental hygienists before they reach an average-proficiency level.

The daily jobs concerning implant treatments were classified into Cluster 3. The intercepts of the item response curves in Cluster 3 were between 0.2 and 0.4. The results showed that fewer than half of the dental hygienists with average-proficiency performed implant treatments.

The daily jobs related to oral care for elderly or hospitalized patients, such as tracheal aspiration, were classified into Cluster 4, and the jobs related to gerodontology were classified into Cluster 5. These jobs provided high item information. The rising phase of the item response curves corresponded to positive ability values. The results showed that the measure of whether these daily jobs are performed is suitable for the discrimination of proficiency levels. However, because Japan is a super-aging society [18], this situation might be unique to Japan.

A variety of jobs were classified into Cluster 6, and the item response curves and item information curves were similar to those of Cluster 3.

The intercepts of the item response curves of Clusters 7 and 8 corresponded to positive ability values. The jobs classified into Cluster 7 were mainly orthodontic treatments, and those classified into Cluster 8 were mainly risk assessments. Training for these daily jobs should target dental hygienists with higher than average-proficiency levels.

The daily jobs related to medical treatments or medical examinations were classified into Cluster 9. The rising phases of the item response curves of Clusters 7 and 8 corresponded to an ability of greater than 1.0, and the probability of a correct response probability against Ability 4 did not reach 0.8. This finding indicated that these jobs require proficiency or high-level skills. Even dental hygienists performing a variety of jobs did not perform these jobs. According to the national survey, 85.3% of dentists worked as a general practitioner at a private dental office in 2016–17, and 90.6% of dental hygienists worked at a dental office [19, 20]. Generally, medical treatments and special oral surgical treatments are not performed in these dental clinics.

The intercept of the curve corresponding to the jobs classified into Cluster 10 exceeded 0.8. This result showed that novices should master these jobs. The probability of a correct answer for probability against Ability −4 was not zero. These jobs may be suitable for undergraduate educational programs.

Jobs classified into Cluster 11 seem to be nonspecialized tasks. These jobs provided little information. As described above, categorizing daily jobs may be helpful for planning lifelong educational programs for dental hygienists.

For the relationship between daily jobs and the motivation to perform the work of dental hygienists, “indirect training of eating function therapy” and “planning of oral care programs for elderly patients” were statistically significantly correlated with the “willingness to work.” Items concerning consultations, including “consultations of treatment plans for patients and their families” and “explanations and consultations of dental treatments,” and teaching, including “management and teaching for staff” and “training and management of dental hygienist students,” were also statistically significantly correlated with the “willingness to work.” If these jobs become routine, the motivation to work may improve. The items “SPT” and “fluoride varnish” classified into Cluster 1 statistically significantly correlated with the “willingness to work.” This result showed that dental hygienists who did not perform these jobs may lose their motivation. In contrast, the “management and ordering of drugs and dental equipment,” which appears to be a daily chore, corresponded to less motivation. Items that made a statistically significant contribution to the “willingness to work” seem to require a relatively high level of proficiency and skills.

“Polishing and adjustment of orthodontic apparatuses” was negatively associated with the “willingness to continue to work.” As shown in Figure 2, the path from daily work to motivation was significant but did not exhibit a strong contribution. In general, motivation can affect a variety of factors other than daily jobs. The results were consistent with those in previous reports [10]. However, performing some specific jobs or not performing these jobs can be strongly associated with the motivation to work.

As specific jobs did not have a strong contribution to motivation, educational programs can be designed according to the clusters presented in this study. Novice or undergraduate dental hygienists should master jobs classified into Clusters 1, 2, and 10. Advanced dental hygienists should master jobs classified into Clusters 3 and 6. Highly advanced dental hygienists should master jobs classified into Clusters 4, 5, and 7. The jobs classified in Clusters 8 and 9 should be considered optional.

## 5. Conclusion

Understanding the features of each daily job of dental hygienists is important for planning dental hygienists' lifelong educational programs and evaluating their skill levels and proficiency levels. The results presented in this study may help to reveal the characteristics of dental hygienists' daily jobs.

## Figures and Tables

**Table 1:** Results of logistic regression analysis for the motivation to work by the work-related task.

	Adjusted odds ratio (95% CI)	*P* value
*Willingness to work*		
Indirect training on eating function therapy	2.791 (1.405–5.544)	0.003
Planning of oral care programs for elderly patients	2.103 (1.218–3.630)	0.008
Consultations for treatment plans for patients and their families	1.779 (1.254–2.526)	0.001
Explanations and consultations regarding dental treatments	1.761 (1.231–2.518)	0.002
SPT	1.697 (1.278–2.255)	<0.001
Management and teaching for staff	1.555 (1.229–1.966)	<0.001
Saliva test	1.528 (1.039–2.248)	0.031
Training and management of dental hygienist students	1.373 (1.013–1.862)	0.041
Fluoride varnish	1.190 (1.046–1.352)	0.008
Management and ordering of drugs and dental equipment	0.496 (0.364–0.675)	<0.001

*Wish to continue to work as a dental hygienist*		
Mobility test of the implant	2.370 (1.502–3.740)	<0.001
Indirect training on eating function therapy	1.881 (1.139–3.104)	0.013
SPT	1.634 (1.083–2.467)	0.019
Oral hygiene instruction for patients for regular checkups	1.211 (1.034–1.418)	0.018
Polishing and adjustment of orthodontic appliances	0.552 (0.363–0.840)	0.005

The responses to “work of a dental hygienist is worthwhile” were provided on a four-point Likert scale: “strongly agree,” “agree,” “disagree,” and “strongly disagree.” For the logistic regression analysis, data were dichotomized as “agree” and “disagree.” For the analysis, all seventy seven items about work-related tasks were used, and significant items were selected by the stepwise method.

## Data Availability

Data will be provided by a reasonable request to the corresponding author after approval of the Japanese Dental Hygienists' Association.

## Abbreviations

SPT: Supportive periodontal therapy
SEM: Structural equation modeling
IRT: Item response theory
MFT: Oral myofunctional therapy.

## Supplementary Materials

Supplementary Materials Table S1: frequencies and item response analysis results of the seventy seven daily jobs of dental hygienists. Table S2: cross tabulations of the work-related tasks by working style and age group. Table S3: results of factor analysis of seventy seven work-related tasks. Figure S1: item response curve and item information curve for seventy-seven items. Figure S2: the mean values of ability of each cluster with respect to age groups. Figure S3: the mean values of ability of each cluster with respect to employment status (full time or part time).
