# Supplementary material for: Prioritization of the Skills to Be Mastered for the Daily Jobs of Japanese Dental Hygienists
Source: Int J Dent. 2020 Jun 22;2020:4297646. doi: 10.1155/2020/4297646 (PMC7327552; doi:10.1155/2020/4297646)
Supplement: Supplementary Materials — Table S1: frequencies and item response analysis results of the seventy seven daily jobs of dental hygienists. Table S2: cross tabulations of the work-related tasks by working style and age group. Table S3: results of factor analysis of seventy seven work-related tasks. Figure S1: item response curve and item information curve for seventy-seven items. Figure S2: the mean values of ability of each cluster with respect to age groups. Figure S3: the mean values of ability of each cluster with respect to employment status (full time or part time). [file 4297646.f1.zip › 4297646.f1/Additinal file 1 S1 Table S2 Table S3 Table.docx]

**Additional file 1**

S1 Table Frequencies and item response analysis results of the seventy-seven daily jobs of dental hygienists

| Cluster |  | Frequency | Three-parameter logistic model | | | Item Information |
| --- | --- | --- | --- | --- | --- | --- |
|  |  |  | Item Discrimination | Item Difficulty | Item Guessing |  |
| 1 | Scaling and root plaining | 3686 (96.8%) | 2.42 | -2.84 | <0.001 | 28.67 |
|  | Oral hygiene instructions for patients with periodontal disease | 3502 (92.0%) | 1.74 | -2.47 | <0.001 | 20.39 |
|  | Periodontal probing | 3569 (93.7%) | 1.70 | -2.50 | <0.001 | 19.81 |
|  | Oral hygiene instructions for pediatric patients | 3238 (85.1%) | 1.30 | -2.03 | <0.001 | 15.08 |
|  | SPT | 3288 (86.4%) | 1.27 | -2.01 | <0.001 | 14.82 |
|  | Oral hygiene instructions for patients for regular check-ups | 3269 (85.9%) | 1.29 | -2.12 | <0.001 | 14.82 |
|  | Fluoride varnish | 3263 (85.7%) | 1.24 | -2.12 | <0.001 | 14.24 |
|  | Impressions for study casts | 3333 (87.5%) | 1.50 | -1.74 | 0.165 | 12.04 |
| 2 | Impressions for occlusal splints | 2519 (66.2%) | 1.59 | -0.66 | <0.001 | 19.79 |
|  | Bite registration for inlay restoration | 2824 (74.2%) | 1.53 | -1.05 | <0.001 | 18.88 |
|  | Bite registration for crown and bridge restoration | 2827 (74.3%) | 1.51 | -1.06 | <0.001 | 18.68 |
|  | Impressions for inlay restoration | 2880 (75.7%) | 1.47 | -1.14 | <0.001 | 18.14 |
|  | Impressions for crown and bridge restoration | 2831 (74.4%) | 1.44 | -1.09 | <0.001 | 17.70 |
|  | Bite registration for occlusal splints | 1958 (51.4%) | 1.32 | -0.09 | <0.001 | 16.34 |
| 3 | Explanations after surgical treatment | 1289 (33.9%) | 1.88 | 0.56 | <0.001 | 23.50 |
|  | Mobility test of the implant | 1231 (32.3%) | 1.66 | 0.65 | <0.001 | 20.60 |
|  | Scaling around the implant | 1441 (37.9%) | 1.62 | 0.42 | <0.001 | 20.12 |
|  | Assistance in implant surgery | 1445 (38.0%) | 1.60 | 0.47 | 0.020 | 18.32 |
| 4 | Tracheal aspiration during feeding training | 44 (1.2%) | 4.65 | 2.35 | 0.004 | 56.84 |
|  | Pharyngeal sputum | 97 (2.5%) | 4.39 | 2.22 | 0.013 | 51.74 |
|  | Masticatory function test | 277 (7.3 %) | 3.85 | 1.99 | 0.045 | 41.14 |
|  | Oral care for hospitalized patients | 835 (21.9%) | 3.74 | 1.67 | 0.170 | 29.77 |
| 5 | Direct training on eating function therapy | 140 (3.7%) | 76.92 | 1.76 | 0.015 | 1449.46 |
|  | Indirect training on eating function therapy | 213 (5.6%) | 63.59 | 1.76 | 0.030 | 974.76 |
|  | Swallowing function test | 343 (9.0%) | 40.77 | 1.76 | 0.059 | 448.50 |
|  | Direct training on swallowing dysfunction | 421 (11.1%) | 43.38 | 1.74 | 0.066 | 428.36 |
|  | Participation in conferences for treatment planning | 433 (11.4%) | 41.27 | 1.74 | 0.069 | 407.59 |
|  | Indirect training on swallowing dysfunction | 680 (17.9%) | 51.15 | 1.72 | 0.125 | 350.16 |
|  | Coordination of medical staff for visiting care | 753 (19.8%) | 43.7 | 1.71 | 0.145 | 325.86 |
|  | Planning of oral care for elderly patients | 567 (14.9%) | 27.05 | 1.71 | 0.097 | 252.37 |
|  | Dental hygiene instructions for elderly or disabled patients by visiting the home | 1269 (33.3%) | 25.22 | 1.61 | 0.288 | 156.62 |
|  | Assistance for dental treatments or oral care for elderly or disabled patients by visiting the home | 1495 (39.3%) | 14.71 | 1.50 | 0.355 | 79.10 |
| 6 | Probing around implants | 1266 (33.3%) | 1.57 | 0.62 | <0.001 | 19.49 |
|  | Gingival retraction for tooth preparation | 1879 (49.4%) | 1.14 | 0.02 | <0.001 | 13.92 |
|  | Impressions for parallel models for orthodontic treatments | 1304 (34.3%) | 1.30 | 0.78 | 0.054 | 13.32 |
|  | Examination of dentition for orthodontic treatments | 1098 (28.8%) | 1.20 | 1.39 | 0.106 | 10.47 |
|  | Checking vitals | 1383 (36.3%) | 1.33 | 1.15 | 0.164 | 10.46 |
|  | Examination of occlusal contact | 1009 (26.5%) | 0.97 | 1.31 | 0.020 | 10.37 |
|  | Checking bite registration | 1115 (29.3%) | 0.91 | 1.13 | 0.010 | 10.11 |
|  | Instructions for fluoride mouthwash | 1660 (43.6%) | 0.82 | 0.29 | <0.001 | 9.56 |
|  | Consultations by telephone | 1014 (26.6%) | 1.18 | 1.76 | 0.133 | 9.41 |
|  | Management and teaching for staff | 2095 (55.0%) | 0.97 | -0.05 | 0.111 | 8.55 |
|  | Pit and fissure sealant | 2286 (60.0%) | 0.74 | -0.76 | <0.001 | 8.28 |
|  | Smoking cessation guidance | 1388 (36.5%) | 0.72 | 0.80 | <0.001 | 7.88 |
| 7 | MFT | 145 (3.8%) | 2.97 | 2.24 | 0.017 | 34.42 |
|  | Monitoring of disabled patients during dental treatments | 251 (6.6%) | 2.70 | 2.13 | 0.036 | 29.37 |
|  | Rubber dam isolation | 270 (7.1%) | 2.11 | 2.17 | 0.029 | 23.08 |
|  | Banding | 241 (6.3%) | 1.80 | 2.21 | 0.011 | 20.60 |
|  | Muscle function training | 451 (11.8%) | 1.87 | 1.83 | 0.035 | 20.15 |
|  | Bracket bonding | 511 (13.4%) | 1.61 | 1.67 | 0.012 | 18.64 |
|  | Polishing and adjustment of orthodontic appliances | 530 (13.9%) | 1.62 | 1.66 | 0.016 | 18.54 |
|  | Removing orthodontic appliances | 658 (17.3%) | 1.62 | 1.53 | 0.035 | 17.42 |
|  | Cephalometric Tracing | 372 (9.8%) | 1.39 | 2.13 | 0.010 | 15.39 |
|  | Installation and monitoring of electrocardiographs | 519 (13.6%) | 1.27 | 2.14 | 0.040 | 12.50 |
| 8 | Malodor test | 385 (10.1%) | 1.18 | 2.46 | 0.020 | 11.58 |
|  | Saliva test | 555 (14.6%) | 1.12 | 2.24 | 0.043 | 10.45 |
|  | Caries risk and activity test | 579 (15.2%) | 1.10 | 2.22 | 0.044 | 10.26 |
|  | Risk assessment of periodontal disease | 455 (12.0%) | 0.97 | 2.37 | <0.001 | 10.05 |
|  | Training and management of dental hygienist students | 867 (22.8%) | 0.69 | 1.91 | <0.001 | 6.84 |
| 9 | Infusion | 48 (1.3%) | 1.52 | 3.65 | 0.003 | 11.96 |
|  | Blood sampling | 52 (1.4%) | 1.36 | 3.73 | <0.001 | 10.30 |
|  | Infusion control | 64 (1.7%) | 1.28 | 3.84 | 0.002 | 8.78 |
|  | Venous access | 30 (0.8%) | 1.30 | 4.28 | <0.001 | 6.88 |
|  | Inhalation sedation | 68 (1.8%) | 1.08 | 4.17 | <0.001 | 6.27 |
| 10 | Prophylactic calculus removal | 3344 (87.8%) | 1.04 | -2.69 | <0.001 | 10.44 |
|  | Assistance in periodontal surgery | 2541 (66.7%) | 1.25 | -0.52 | 0.145 | 10.42 |
|  | Instructions for handling dentures | 3459 (90.9%) | 1.50 | -2.23 | 0.242 | 10.14 |
|  | Explanations of precautions for pre- and post-surgical treatments | 3207 (84.2%) | 1.50 | -1.30 | 0.272 | 9.63 |
|  | Counseling for medication | 3026 (79.5%) | 0.84 | -1.98 | <0.001 | 8.88 |
|  | Explanations and consultations regarding general dental treatments | 3438 (90.3%) | 1.73 | -1.49 | 0.450 | 7.50 |
|  | Preliminary oral examination | 3549 (93.2%) | 1.70 | -1.85 | 0.457 | 7.22 |
| 11 | Management and ordering of drugs and dental equipment | 2961 (77.8%) | 0.55 | -2.69 | <0.001 | 4.51 |
|  | Consultations of treatment plans for patients and their families | 3415 (89.7%) | 1.45 | -1.23 | 0.584 | 4.45 |
|  | Accounting | 1071 (28.1%) | 0.53 | 2.36 | 0.070 | 3.50 |
|  | Inputting data in a computer | 1899 (49.9%) | 0.50 | 0.64 | 0.157 | 3.21 |
|  | Sterilization and disinfection of dental equipment | 3489 (91.6%) | 0.54 | -5.37 | 0.001 | 2.17 |
|  | Reception work | 2113 (55.5%) | 0.25 | -1.08 | 0.011 | 1.40 |

Jobs were clustered according to the item response curve.

S2 Table cross tabulations of the work-related tasks by working style and age group

|  | | Regular employee | Part time worker | P-value | Age group | | | | | | | | | | P-value |
| --- | --- | --- | --- | --- | --- | --- | --- | --- | --- | --- | --- | --- | --- | --- | --- |
|  |  |  |  |  | <24 | 25-29 | 30-34 | 35-39 | 40-44 | 45-49 | 50-54 | 55-60 | 60-64 | 64< |  |
| Preliminary examination of the oral cavity | - | 106 | 108 | 0.021 | 24 | 13 | 17 | 20 | 33 | 32 | 44 | 20 | 7 | 5 | 0.435 |
|  | + | 2014 | 1485 |  | 384 | 308 | 282 | 369 | 506 | 655 | 544 | 303 | 128 | 43 |  |
| Explanations and consultations regarding treatment | - | 141 | 172 | <0.001 | 32 | 17 | 26 | 28 | 44 | 64 | 57 | 26 | 10 | 9 | 0.102 |
|  | + | 1978 | 1411 |  | 376 | 304 | 273 | 361 | 494 | 621 | 532 | 292 | 122 | 37 |  |
| Explanations of preoperative precautions | - | 249 | 282 | <0.001 | 55 | 40 | 30 | 46 | 80 | 119 | 96 | 38 | 16 | 11 | 0.015 |
|  | + | 1867 | 1296 |  | 352 | 280 | 269 | 343 | 458 | 562 | 489 | 280 | 116 | 36 |  |
| Medication counseling | - | 333 | 364 | <0.001 | 84 | 52 | 47 | 50 | 114 | 134 | 126 | 63 | 17 | 12 | 0.005 |
|  | + | 1774 | 1208 |  | 323 | 269 | 252 | 339 | 421 | 545 | 455 | 253 | 114 | 33 |  |
| Consultations with patients and their families | - | 152 | 179 | <0.001 | 32 | 20 | 26 | 27 | 56 | 61 | 63 | 26 | 12 | 7 | 0.226 |
|  | + | 1963 | 1405 |  | 376 | 301 | 273 | 362 | 482 | 622 | 522 | 292 | 123 | 39 |  |
| Checking vitals | - | 1229 | 1066 | <0.001 | 253 | 190 | 178 | 241 | 344 | 441 | 356 | 204 | 70 | 26 | 0.435 |
|  | + | 865 | 491 |  | 153 | 130 | 120 | 143 | 191 | 230 | 223 | 111 | 53 | 19 |  |
| Installation of electrocardiographs and monitor | - | 1716 | 1373 | <0.001 | 347 | 266 | 242 | 316 | 453 | 594 | 489 | 264 | 98 | 38 | 0.014 |
|  | + | 341 | 168 |  | 58 | 52 | 55 | 66 | 76 | 63 | 77 | 45 | 16 | 5 |  |
| Saliva test | - | 1691 | 1353 | <0.001 | 324 | 258 | 246 | 330 | 451 | 570 | 482 | 263 | 102 | 37 | 0.119 |
|  | + | 362 | 185 |  | 80 | 59 | 49 | 52 | 71 | 89 | 84 | 43 | 15 | 7 |  |
| Caries risk and activity test | - | 1701 | 1333 | 0.003 | 324 | 249 | 249 | 331 | 452 | 575 | 482 | 256 | 100 | 32 | 0.003 |
|  | + | 356 | 210 |  | 82 | 69 | 47 | 54 | 75 | 88 | 82 | 47 | 16 | 12 |  |
| Risk assessment of periodontal disease | - | 1792 | 1354 | 0.771 | 358 | 277 | 265 | 341 | 477 | 585 | 482 | 254 | 88 | 35 | <0.001 |
|  | + | 255 | 187 |  | 47 | 40 | 31 | 40 | 50 | 74 | 79 | 50 | 29 | 9 |  |
| Malodor test | - | 1825 | 1393 | 0.240 | 369 | 286 | 265 | 343 | 469 | 594 | 496 | 272 | 99 | 38 | 0.741 |
|  | + | 224 | 150 |  | 36 | 32 | 30 | 42 | 58 | 63 | 64 | 34 | 18 | 6 |  |
| Occlusal contact test | - | 1427 | 1184 | <0.001 | 268 | 206 | 203 | 267 | 393 | 499 | 441 | 232 | 82 | 36 | 0.000 |
|  | + | 638 | 357 |  | 138 | 112 | 95 | 114 | 135 | 165 | 125 | 74 | 34 | 9 |  |
| Checking bite | - | 1394 | 1113 | 0.005 | 257 | 207 | 207 | 259 | 371 | 490 | 416 | 210 | 75 | 29 | 0.005 |
|  | + | 664 | 431 |  | 147 | 111 | 90 | 123 | 160 | 167 | 154 | 95 | 42 | 16 |  |
| Examination of dentition | - | 1395 | 1127 | 0.001 | 263 | 207 | 199 | 279 | 380 | 473 | 410 | 220 | 76 | 26 | 0.016 |
|  | + | 662 | 415 |  | 141 | 110 | 97 | 104 | 149 | 181 | 156 | 88 | 43 | 19 |  |
| Masticatory function test | - | 1904 | 1407 | 0.090 | 378 | 294 | 279 | 354 | 496 | 597 | 516 | 277 | 101 | 39 | 0.287 |
|  | + | 142 | 130 |  | 28 | 22 | 17 | 27 | 30 | 60 | 47 | 25 | 13 | 5 |  |
| Swallowing function test | - | 1869 | 1385 | 0.289 | 373 | 295 | 274 | 358 | 484 | 585 | 496 | 271 | 99 | 40 | 0.004 |
|  | + | 184 | 154 |  | 33 | 23 | 22 | 24 | 40 | 72 | 68 | 37 | 18 | 3 |  |
| Probing | - | 87 | 99 | 0.003 | 18 | 7 | 12 | 20 | 32 | 34 | 34 | 18 | 9 | 8 | 0.006 |
|  | + | 2035 | 1486 |  | 391 | 314 | 287 | 369 | 507 | 649 | 556 | 304 | 125 | 38 |  |
| Scaling and root planing | - | 32 | 39 | 0.036 | 4 | 5 | 2 | 8 | 14 | 12 | 15 | 5 | 4 | 5 | 0.001 |
|  | + | 2089 | 1546 |  | 403 | 316 | 297 | 381 | 525 | 673 | 575 | 316 | 130 | 41 |  |
| SPT | - | 203 | 249 | <0.001 | 42 | 31 | 28 | 43 | 60 | 86 | 93 | 42 | 17 | 13 | 0.003 |
|  | + | 1914 | 1326 |  | 366 | 290 | 271 | 345 | 479 | 596 | 494 | 277 | 111 | 33 |  |
| Assist in periodontal surgery | - | 583 | 591 | <0.001 | 122 | 94 | 94 | 120 | 165 | 214 | 200 | 111 | 44 | 15 | 0.722 |
|  | + | 1528 | 974 |  | 286 | 227 | 204 | 267 | 372 | 467 | 385 | 206 | 80 | 27 |  |
| Study model impressions | - | 166 | 240 | <0.001 | 39 | 28 | 24 | 37 | 55 | 69 | 84 | 46 | 23 | 9 | 0.002 |
|  | + | 1946 | 1344 |  | 370 | 293 | 274 | 350 | 483 | 614 | 506 | 272 | 109 | 36 |  |
| Gingival retraction for tooth preparation | - | 965 | 859 | <0.001 | 233 | 153 | 135 | 187 | 248 | 351 | 293 | 151 | 62 | 24 | 0.051 |
|  | + | 1136 | 713 |  | 174 | 167 | 163 | 200 | 288 | 326 | 292 | 162 | 68 | 21 |  |
| Impressions of inlay cavity | - | 411 | 437 | <0.001 | 75 | 58 | 55 | 80 | 111 | 175 | 148 | 91 | 44 | 18 | <0.001 |
|  | + | 1695 | 1144 |  | 334 | 262 | 242 | 306 | 427 | 505 | 441 | 225 | 88 | 27 |  |
| Bite assessment for inlay cavities | - | 437 | 458 | <0.001 | 86 | 61 | 56 | 77 | 126 | 173 | 164 | 90 | 52 | 19 | <0.001 |
|  | + | 1665 | 1120 |  | 323 | 258 | 241 | 310 | 412 | 503 | 424 | 226 | 78 | 26 |  |
| Impressions for occlusal splints | - | 566 | 616 | <0.001 | 139 | 76 | 62 | 101 | 147 | 236 | 211 | 126 | 67 | 24 | <0.001 |
|  | + | 1531 | 948 |  | 267 | 245 | 235 | 285 | 384 | 440 | 374 | 187 | 61 | 20 |  |
| Bite assessments for occlusal splints | - | 927 | 795 | <0.001 | 205 | 144 | 111 | 156 | 222 | 331 | 304 | 156 | 74 | 26 | <0.001 |
|  | + | 1156 | 766 |  | 200 | 176 | 184 | 228 | 307 | 340 | 281 | 155 | 51 | 18 |  |
| Impressions for crowns or bridges | - | 428 | 464 | <0.001 | 83 | 62 | 57 | 77 | 117 | 183 | 156 | 100 | 48 | 18 | <0.001 |
|  | + | 1677 | 1115 |  | 326 | 257 | 240 | 310 | 421 | 496 | 432 | 216 | 83 | 27 |  |
| Occlusal assessments for crowns or bridges | - | 436 | 457 | <0.001 | 84 | 60 | 58 | 72 | 128 | 174 | 163 | 91 | 53 | 19 | <0.001 |
|  | + | 1667 | 1121 |  | 325 | 261 | 239 | 315 | 410 | 503 | 425 | 224 | 76 | 26 |  |
| Trace of cephalometry | - | 1810 | 1359 | 0.115 | 348 | 276 | 253 | 332 | 468 | 598 | 501 | 269 | 109 | 35 | 0.006 |
|  | + | 223 | 140 |  | 57 | 39 | 39 | 49 | 47 | 55 | 52 | 19 | 8 | 3 |  |
| Banding | - | 1881 | 1396 | 0.515 | 376 | 289 | 269 | 351 | 470 | 613 | 507 | 279 | 109 | 36 | 0.190 |
|  | + | 140 | 95 |  | 28 | 24 | 21 | 30 | 41 | 35 | 41 | 8 | 7 | 2 |  |
| Bracket bonding | - | 1710 | 1318 | 0.001 | 330 | 257 | 251 | 326 | 432 | 572 | 479 | 261 | 103 | 35 | 0.008 |
|  | + | 322 | 178 |  | 74 | 58 | 40 | 55 | 83 | 79 | 75 | 26 | 14 | 3 |  |
| Impressions of jaws for parallel models | - | 1210 | 1050 | <0.001 | 221 | 174 | 166 | 238 | 328 | 449 | 376 | 211 | 80 | 31 | <0.001 |
|  | + | 835 | 451 |  | 185 | 142 | 126 | 142 | 188 | 208 | 180 | 80 | 37 | 8 |  |
| Polishing and adjustment of orthodontic apparatuses | - | 1696 | 1319 | <0.001 | 321 | 260 | 251 | 326 | 436 | 568 | 480 | 258 | 99 | 35 | 0.011 |
|  | + | 340 | 179 |  | 84 | 55 | 41 | 56 | 78 | 85 | 73 | 31 | 18 | 3 |  |
| Removal of orthodontic apparatuses | - | 1619 | 1268 | <0.001 | 311 | 253 | 234 | 304 | 407 | 548 | 468 | 251 | 96 | 35 | 0.003 |
|  | + | 418 | 231 |  | 94 | 62 | 58 | 78 | 110 | 104 | 86 | 37 | 21 | 3 |  |
| Muscle function training | - | 1742 | 1336 | 0.001 | 341 | 268 | 249 | 335 | 446 | 583 | 480 | 264 | 99 | 33 | 0.022 |
|  | + | 286 | 156 |  | 63 | 48 | 42 | 45 | 70 | 60 | 73 | 23 | 18 | 5 |  |
| Probing of implants | - | 1250 | 1086 | <0.001 | 247 | 178 | 172 | 235 | 339 | 465 | 382 | 210 | 86 | 35 | <0.001 |
|  | + | 807 | 436 |  | 159 | 138 | 125 | 147 | 185 | 194 | 180 | 90 | 34 | 4 |  |
| Mobility test of the implant | - | 1261 | 1108 | <0.001 | 249 | 168 | 165 | 240 | 343 | 463 | 399 | 226 | 92 | 36 | <0.001 |
|  | + | 796 | 411 |  | 157 | 148 | 131 | 143 | 181 | 196 | 160 | 74 | 28 | 3 |  |
| Scaling around the implant | - | 1130 | 1027 | <0.001 | 228 | 151 | 154 | 210 | 311 | 443 | 359 | 196 | 86 | 32 | <0.001 |
|  | + | 923 | 493 |  | 177 | 164 | 140 | 171 | 212 | 216 | 202 | 104 | 36 | 7 |  |
| Assistance in implant surgery | - | 1117 | 1042 | <0.001 | 224 | 159 | 142 | 215 | 311 | 424 | 370 | 210 | 86 | 32 | <0.001 |
|  | + | 945 | 476 |  | 181 | 158 | 153 | 168 | 213 | 236 | 192 | 88 | 36 | 8 |  |
| Explanations after the operation | - | 1216 | 1093 | <0.001 | 265 | 176 | 160 | 234 | 333 | 453 | 377 | 209 | 85 | 32 | <0.001 |
|  | + | 843 | 424 |  | 141 | 140 | 135 | 148 | 191 | 206 | 186 | 89 | 36 | 7 |  |
| Infusion control | - | 2004 | 1499 | 0.003 | 397 | 313 | 290 | 372 | 515 | 643 | 543 | 291 | 120 | 40 | 0.785 |
|  | + | 47 | 15 |  | 7 | 4 | 6 | 9 | 9 | 12 | 13 | 4 | 0 | 0 |  |
| Venous access | - | 2029 | 1506 | 0.045 | 400 | 315 | 294 | 375 | 518 | 652 | 549 | 294 | 120 | 40 | 0.583 |
|  | + | 22 | 7 |  | 4 | 2 | 2 | 6 | 5 | 3 | 7 | 1 | 0 | 0 |  |
| Blood sampling | - | 2020 | 1498 | 0.198 | 397 | 312 | 293 | 373 | 517 | 649 | 548 | 294 | 116 | 40 | 0.391 |
|  | + | 32 | 16 |  | 7 | 5 | 3 | 8 | 8 | 6 | 8 | 1 | 4 | 0 |  |
| Infusion | - | 2023 | 1496 | 0.605 | 399 | 312 | 292 | 371 | 516 | 648 | 549 | 293 | 120 | 40 | 0.345 |
|  | + | 27 | 17 |  | 5 | 4 | 4 | 10 | 8 | 7 | 7 | 1 | 0 | 0 |  |
| Inhalation sedation | - | 2010 | 1485 | 0.992 | 395 | 312 | 290 | 370 | 511 | 644 | 545 | 293 | 117 | 40 | 0.710 |
|  | + | 38 | 28 |  | 9 | 5 | 6 | 11 | 11 | 10 | 11 | 2 | 2 | 0 |  |
| Monitoring during general dental treatment | - | 1856 | 1376 | 0.643 | 376 | 294 | 270 | 355 | 464 | 604 | 496 | 255 | 101 | 35 | 0.041 |
|  | + | 145 | 101 |  | 28 | 17 | 19 | 24 | 40 | 31 | 42 | 30 | 14 | 4 |  |
| Rubber dam | - | 1835 | 1386 | 0.031 | 363 | 284 | 264 | 347 | 469 | 607 | 498 | 264 | 104 | 38 | 0.119 |
|  | + | 169 | 96 |  | 41 | 27 | 24 | 32 | 37 | 30 | 43 | 21 | 12 | 2 |  |
| Eating function therapy, indirect training | - | 1905 | 1371 | 0.004 | 391 | 302 | 283 | 355 | 481 | 599 | 490 | 258 | 100 | 36 | <0.001 |
|  | + | 99 | 108 |  | 12 | 9 | 6 | 24 | 25 | 36 | 50 | 28 | 16 | 4 |  |
| Eating function therapy, direct training | - | 1931 | 1413 | 0.200 | 395 | 301 | 283 | 364 | 490 | 608 | 512 | 266 | 106 | 39 | 0.007 |
|  | + | 71 | 65 |  | 8 | 10 | 6 | 15 | 15 | 26 | 28 | 20 | 9 | 1 |  |
| MFT | - | 1915 | 1423 | 0.451 | 386 | 299 | 278 | 363 | 491 | 615 | 507 | 271 | 108 | 40 | 0.247 |
|  | + | 86 | 56 |  | 16 | 12 | 11 | 16 | 15 | 20 | 33 | 14 | 6 | 0 |  |
| Tracheal aspiration of the trachea during feeding training | - | 1985 | 1453 | 0.016 | 401 | 310 | 287 | 374 | 501 | 624 | 531 | 280 | 109 | 40 | 0.061 |
|  | + | 16 | 25 |  | 2 | 1 | 2 | 5 | 5 | 10 | 8 | 5 | 5 | 0 |  |
| Pharyngeal sputum | - | 1957 | 1426 | 0.087 | 395 | 306 | 285 | 367 | 495 | 615 | 523 | 271 | 105 | 40 | 0.003 |
|  | + | 45 | 47 |  | 8 | 4 | 4 | 11 | 11 | 19 | 16 | 12 | 10 | 0 |  |
| Assistance in dental visits in home care | - | 1174 | 919 | 0.120 | 268 | 204 | 196 | 230 | 305 | 360 | 291 | 172 | 59 | 20 | <0.001 |
|  | + | 863 | 607 |  | 137 | 108 | 97 | 148 | 207 | 296 | 268 | 127 | 68 | 24 |  |
| Dental hygiene instructions by visiting patients at home | - | 1331 | 979 | 0.478 | 298 | 225 | 214 | 256 | 341 | 405 | 312 | 181 | 67 | 22 | <0.001 |
|  | + | 702 | 543 |  | 107 | 87 | 79 | 122 | 171 | 248 | 246 | 115 | 60 | 22 |  |
| Feeding. Indirect training on swallowing dysfunction | - | 1698 | 1177 | <0.001 | 374 | 280 | 258 | 319 | 422 | 501 | 405 | 219 | 86 | 29 | <0.001 |
|  | + | 326 | 339 |  | 31 | 32 | 34 | 59 | 89 | 146 | 150 | 76 | 38 | 14 |  |
| Feeding. Direct training on swallowing dysfunction | - | 1815 | 1300 | <0.001 | 386 | 292 | 268 | 339 | 450 | 554 | 462 | 250 | 99 | 33 | <0.001 |
|  | + | 202 | 210 |  | 19 | 20 | 24 | 39 | 60 | 89 | 91 | 44 | 20 | 10 |  |
| Oral care plan making | - | 1746 | 1234 | <0.001 | 376 | 283 | 259 | 328 | 442 | 521 | 433 | 233 | 89 | 31 | <0.001 |
|  | + | 273 | 281 |  | 29 | 29 | 33 | 50 | 69 | 125 | 120 | 61 | 34 | 11 |  |
| Participation in conferences | - | 1809 | 1297 | <0.001 | 381 | 290 | 266 | 338 | 458 | 563 | 452 | 242 | 101 | 33 | <0.001 |
|  | + | 208 | 216 |  | 24 | 22 | 26 | 39 | 52 | 81 | 101 | 53 | 21 | 9 |  |
| Adjustments with medical staff | - | 1611 | 1191 | 0.432 | 355 | 262 | 246 | 299 | 414 | 498 | 403 | 221 | 88 | 31 | <0.001 |
|  | + | 413 | 326 |  | 50 | 49 | 46 | 78 | 98 | 150 | 153 | 75 | 36 | 11 |  |
| Fluoride varnish | - | 181 | 229 | <0.001 | 25 | 22 | 20 | 40 | 52 | 83 | 82 | 58 | 18 | 12 | <0.001 |
|  | + | 1902 | 1319 |  | 381 | 294 | 274 | 344 | 473 | 592 | 492 | 254 | 104 | 31 |  |
| Fissure sealant | - | 712 | 639 | <0.001 | 138 | 87 | 85 | 141 | 170 | 269 | 268 | 125 | 56 | 21 | <0.001 |
|  | + | 1359 | 899 |  | 265 | 230 | 210 | 242 | 350 | 401 | 303 | 183 | 65 | 20 |  |
| Prophylactic calculus removal except for periodontal treatment. | - | 148 | 175 | <0.001 | 14 | 22 | 11 | 24 | 37 | 55 | 84 | 51 | 20 | 7 | <0.001 |
|  | + | 1930 | 1369 |  | 392 | 293 | 284 | 360 | 484 | 619 | 486 | 262 | 104 | 35 |  |
| Dental hygiene practical guidance, pediatric patients | - | 191 | 235 | <0.001 | 26 | 24 | 20 | 45 | 55 | 89 | 84 | 53 | 22 | 10 | <0.001 |
|  | + | 1888 | 1306 |  | 378 | 291 | 275 | 340 | 471 | 585 | 485 | 258 | 98 | 33 |  |
| Dental hygiene practical guidance, patients with periodontal disease | - | 86 | 102 | 0.001 | 17 | 13 | 13 | 16 | 26 | 41 | 35 | 20 | 7 | 3 | 0.743 |
|  | + | 2004 | 1451 |  | 389 | 303 | 282 | 369 | 502 | 634 | 540 | 294 | 118 | 42 |  |
| Patient guidance by recall | - | 175 | 219 | <0.001 | 26 | 15 | 22 | 27 | 50 | 84 | 85 | 59 | 21 | 9 | <0.001 |
|  | + | 1904 | 1324 |  | 378 | 299 | 271 | 355 | 475 | 590 | 488 | 250 | 103 | 36 |  |
| Guidance on handling dentures | - | 108 | 118 | 0.002 | 29 | 22 | 16 | 23 | 28 | 46 | 37 | 18 | 7 | 3 | 0.978 |
|  | + | 1983 | 1430 |  | 376 | 294 | 278 | 362 | 496 | 630 | 539 | 296 | 119 | 42 |  |
| Instructions on fluoride mouthwash | - | 1094 | 886 | 0.004 | 236 | 171 | 161 | 191 | 285 | 391 | 314 | 160 | 54 | 26 | 0.083 |
|  | + | 981 | 653 |  | 168 | 145 | 133 | 193 | 241 | 277 | 257 | 147 | 65 | 19 |  |
| Smoking cessation guidance | - | 1233 | 1009 | 0.000 | 265 | 187 | 167 | 236 | 343 | 431 | 348 | 184 | 69 | 24 | 0.273 |
|  | + | 840 | 526 |  | 140 | 129 | 124 | 146 | 184 | 240 | 222 | 124 | 50 | 16 |  |
| Oral care for hospitalized patients | - | 1580 | 1185 | 0.518 | 320 | 250 | 231 | 291 | 409 | 510 | 413 | 240 | 85 | 30 | 0.397 |
|  | + | 479 | 341 |  | 85 | 65 | 60 | 91 | 116 | 151 | 150 | 67 | 33 | 8 |  |
| Consultations by telephone | - | 1431 | 1177 | 0.000 | 296 | 211 | 204 | 275 | 385 | 501 | 418 | 226 | 78 | 26 | 0.259 |
|  | + | 642 | 355 |  | 109 | 102 | 89 | 106 | 142 | 170 | 151 | 82 | 41 | 14 |  |
| Reception office | - | 799 | 774 | 0.000 | 175 | 120 | 121 | 135 | 231 | 314 | 262 | 145 | 50 | 20 | 0.021 |
|  | + | 1295 | 781 |  | 233 | 197 | 175 | 248 | 298 | 364 | 316 | 168 | 73 | 25 |  |
| Accounting | - | 1387 | 1198 | 0.000 | 293 | 228 | 212 | 263 | 377 | 492 | 406 | 215 | 73 | 27 | 0.305 |
|  | + | 697 | 347 |  | 113 | 89 | 84 | 118 | 150 | 182 | 167 | 97 | 47 | 16 |  |
| Inputting data in a computer | - | 894 | 872 | 0.000 | 208 | 132 | 127 | 170 | 258 | 354 | 280 | 153 | 56 | 25 | 0.028 |
|  | + | 1184 | 676 |  | 198 | 184 | 165 | 212 | 268 | 318 | 297 | 157 | 66 | 19 |  |
| Equipment, sterilization and disinfection of dental equipment | - | 78 | 135 | 0.000 | 13 | 13 | 11 | 15 | 32 | 48 | 42 | 24 | 12 | 6 | 0.003 |
|  | + | 2019 | 1425 |  | 395 | 304 | 285 | 368 | 498 | 632 | 537 | 291 | 113 | 39 |  |
| Management and ordering of drugs and dental equipment | - | 232 | 499 | 0.000 | 66 | 30 | 41 | 48 | 98 | 164 | 140 | 90 | 38 | 16 | 0.000 |
|  | + | 1862 | 1056 |  | 342 | 287 | 255 | 334 | 432 | 515 | 439 | 223 | 85 | 27 |  |
| Staff teaching management | - | 726 | 843 | 0.000 | 234 | 116 | 102 | 151 | 213 | 307 | 244 | 135 | 46 | 22 | 0.000 |
|  | + | 1356 | 702 |  | 172 | 200 | 193 | 230 | 313 | 370 | 331 | 174 | 73 | 21 |  |
| Trainee management of dental hygienist students | - | 1501 | 1263 | 0.000 | 298 | 226 | 216 | 286 | 381 | 550 | 446 | 252 | 91 | 32 | 0.001 |
|  | + | 576 | 277 |  | 109 | 89 | 77 | 95 | 147 | 122 | 127 | 56 | 28 | 10 |  |

S3 Table Results of factor analysis of seventy-seven work-related tasks

|  | Factor | | |
| --- | --- | --- | --- |
|  | 1 | 2 | 3 |
| Direct training on eating function therapy | 0.945 | -0.003 | 0.014 |
| Indirect training on eating function therapy | 0.813 | 0.020 | 0.010 |
| Planning of oral care program for elderly patients | 0.423 | 0.023 | 0.053 |
| Probing around implants | 0.021 | 0.811 | 0.046 |
| Assistance in implant surgery | -0.006 | 0.728 | 0.022 |
| Oral hygiene instructions for patients for regular check-ups | 0.003 | 0.326 | 0.182 |
| Management and teaching for staff | 0.044 | 0.274 | 0.211 |
| Explanations and consultations regarding dental treatment | 0.030 | 0.123 | 0.711 |
| Consultations for treatment plans for patients and their families | 0.045 | 0.104 | 0.664 |
| Total | 1.737 | 1.397 | 1.029 |
| Percent of variance | 19.304 | 15.524 | 11.435 |
| Cumulative percent | 19.304 | 34.828 | 46.263 |
