# Supplementary material for: Prioritization of the Skills to Be Mastered for the Daily Jobs of Japanese Dental Hygienists
Source: Int J Dent. 2020 Jun 22;2020:4297646. doi: 10.1155/2020/4297646 (PMC7327552; doi:10.1155/2020/4297646)
Supplement: Supplementary Materials — Table S1: frequencies and item response analysis results of the seventy seven daily jobs of dental hygienists. Table S2: cross tabulations of the work-related tasks by working style and age group. Table S3: results of factor analysis of seventy seven work-related tasks. Figure S1: item response curve and item information curve for seventy-seven items. Figure S2: the mean values of ability of each cluster with respect to age groups. Figure S3: the mean values of ability of each cluster with respect to employment status (full time or part time). [file 4297646.f1.zip › 4297646.f1/Additional file 3 S2 Figuer .pptx]

## Slide 1
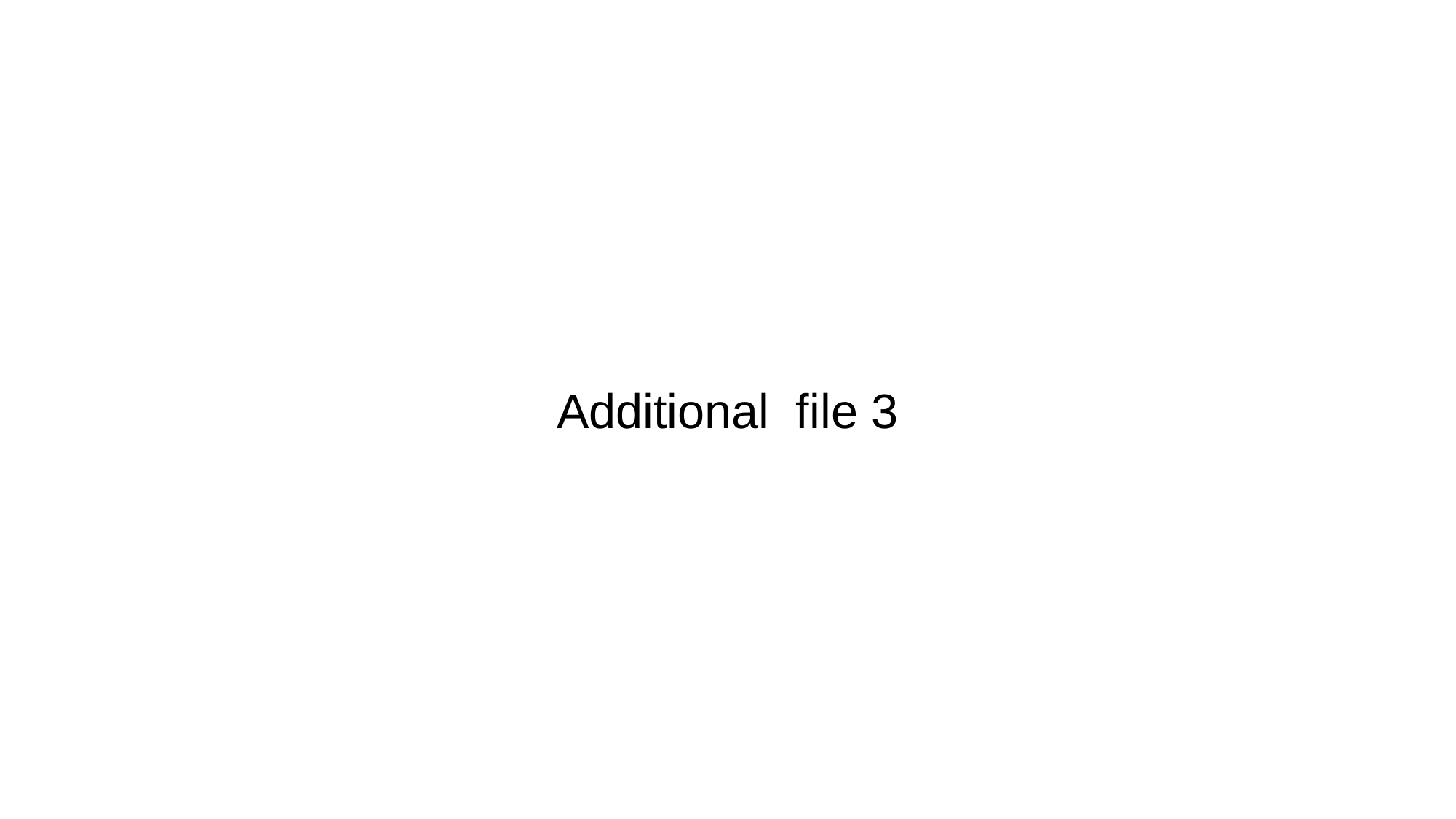

Additional file 3

## Slide 2
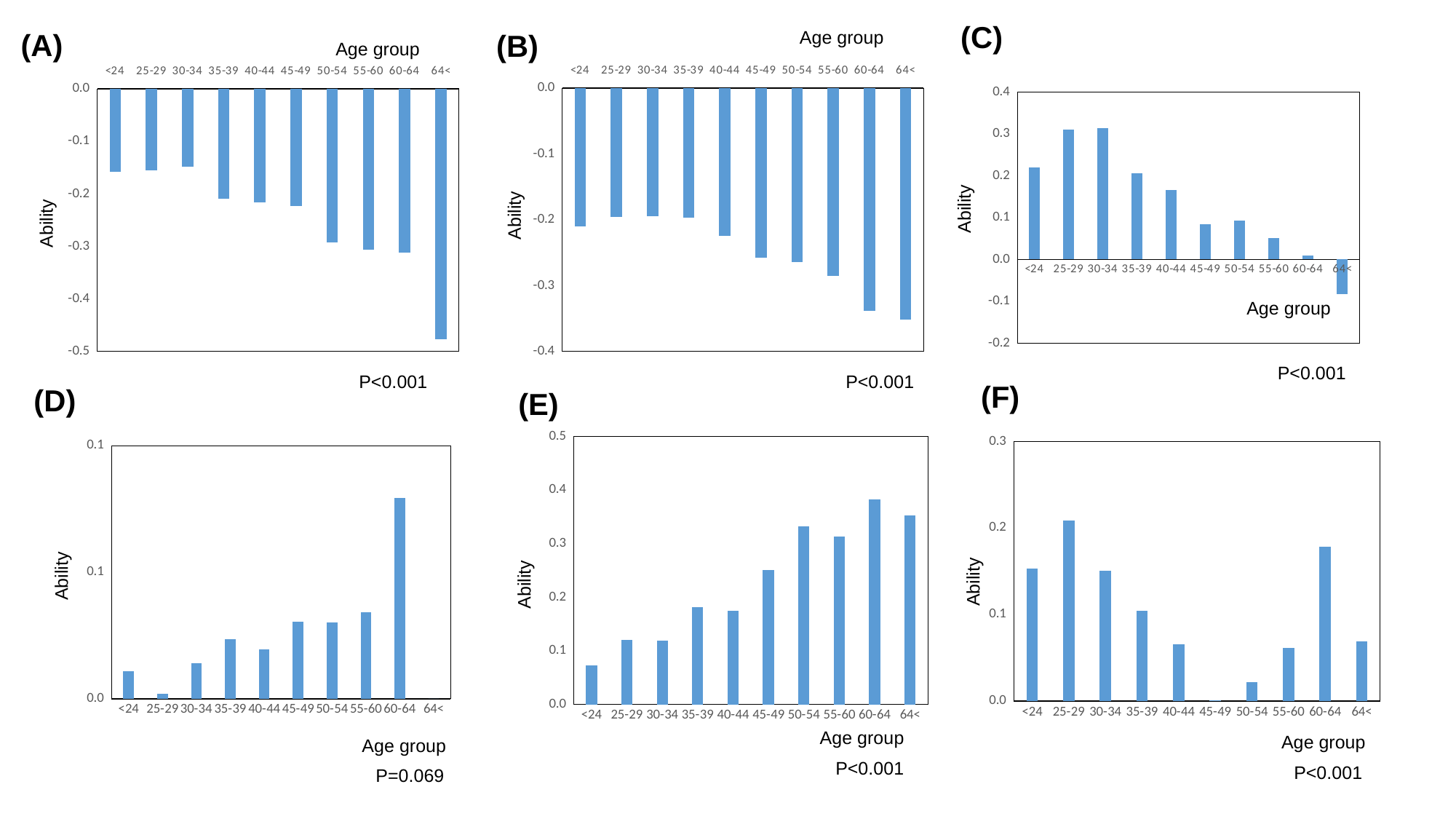

(C)
Age group
(A)
(B)
Age group
### Chart
| Category | |
|---|---|
| <24 | -0.21052682926829258 |
| 25-29 | -0.19599380804953553 |
| 30-34 | -0.19434666666666656 |
| 35-39 | -0.19703316326530593 |
| 40-44 | -0.22418749999999993 |
| 45-49 | -0.25771428571428534 |
| 50-54 | -0.26378787878787857 |
| 55-60 | -0.2847865853658535 |
| 60-64 | -0.33782269503546086 |
| 64< | -0.3519811320754717 |
### Chart
| Category | |
|---|---|
| <24 | -0.15768536585365742 |
| 25-29 | -0.15588854489163972 |
| 30-34 | -0.14894666666666606 |
| 35-39 | -0.20980102040816181 |
| 40-44 | -0.21608823529411625 |
| 45-49 | -0.22240692640692586 |
| 50-54 | -0.29261279461279516 |
| 55-60 | -0.3063384146341456 |
| 60-64 | -0.3122624113475175 |
| 64< | -0.47720754716981134 |
### Chart
| Category | |
|---|---|
| <24 | 0.2206487804878046 |
| 25-29 | 0.30980804953560354 |
| 30-34 | 0.3136766666666666 |
| 35-39 | 0.2065331632653058 |
| 40-44 | 0.1652205882352937 |
| 45-49 | 0.08356854256854197 |
| 50-54 | 0.09284680134680087 |
| 55-60 | 0.05099085365853611 |
| 60-64 | 0.009588652482269585 |
| 64< | -0.08305660377358491 |Ability
Ability
Ability
Age group
P<0.001
P<0.001
P<0.001
(F)
(D)
(E)
### Chart
| Category | |
|---|---|
| <24 | 0.010921951219512194 |
| 25-29 | 0.002021671826625387 |
| 30-34 | 0.014033333333333333 |
| 35-39 | 0.02350765306122449 |
| 40-44 | 0.019593749999999997 |
| 45-49 | 0.030493506493506496 |
| 50-54 | 0.03015488215488216 |
| 55-60 | 0.03413109756097561 |
| 60-64 | 0.07939716312056738 |
| 64< | 0.0 |
### Chart
| Category | |
|---|---|
| <24 | 0.07289756097561013 |
| 25-29 | 0.12008978328173342 |
| 30-34 | 0.11893000000000001 |
| 35-39 | 0.18119897959183565 |
| 40-44 | 0.17469301470588325 |
| 45-49 | 0.25078066378066527 |
| 50-54 | 0.3321414141414155 |
| 55-60 | 0.3140731707317079 |
| 60-64 | 0.38263829787234005 |
| 64< | 0.35226415094339614 |
### Chart
| Category | |
|---|---|
| <24 | 0.15356341463414638 |
| 25-29 | 0.20886068111455106 |
| 30-34 | 0.15090333333333333 |
| 35-39 | 0.10413775510204092 |
| 40-44 | 0.06579227941176484 |
| 45-49 | 0.0010490620490620486 |
| 50-54 | 0.022104377104377154 |
| 55-60 | 0.06160365853658556 |
| 60-64 | 0.17804255319148946 |
| 64< | 0.0688867924528302 |Ability
Ability
Ability
Age group
Age group
Age group
P<0.001
P<0.001
P=0.069

## Slide 3
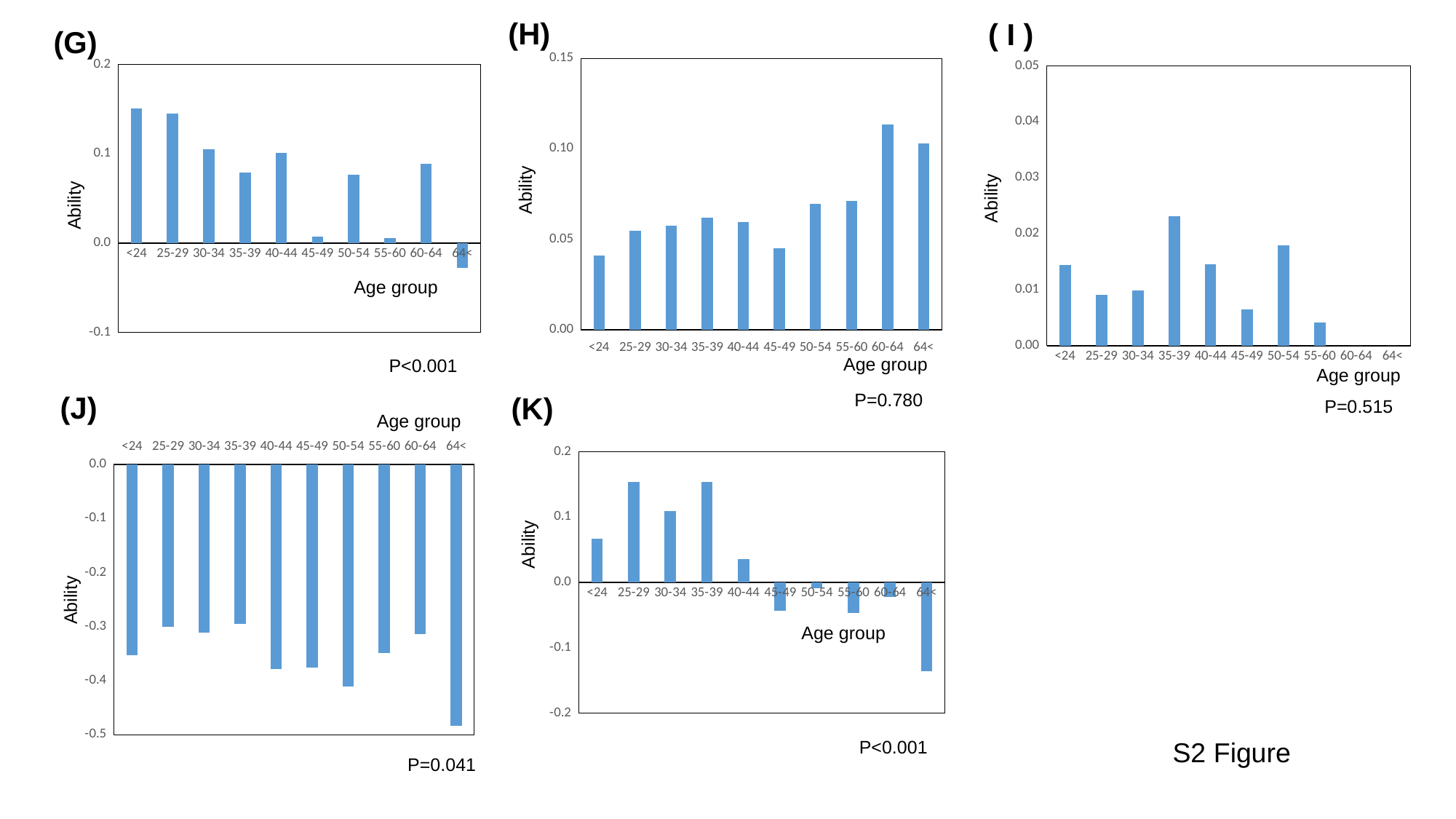

(H)
( I )
(G)
### Chart
| Category | |
|---|---|
| <24 | 0.040943902439024286 |
| 25-29 | 0.05462229102167092 |
| 30-34 | 0.05744666666666649 |
| 35-39 | 0.06188265306122398 |
| 40-44 | 0.05967647058823439 |
| 45-49 | 0.045220779220778724 |
| 50-54 | 0.06947811447811429 |
| 55-60 | 0.07127134146341407 |
| 60-64 | 0.11353900709219854 |
| 64< | 0.10300000000000005 |
### Chart
| Category | |
|---|---|
| <24 | 0.15030243902438967 |
| 25-29 | 0.14491331269349855 |
| 30-34 | 0.1051566666666671 |
| 35-39 | 0.07920153061224554 |
| 40-44 | 0.1012867647058818 |
| 45-49 | 0.007363636363636308 |
| 50-54 | 0.07652525252525219 |
| 55-60 | 0.005493902439024373 |
| 60-64 | 0.08877304964539004 |
| 64< | -0.02779245283018869 |
### Chart
| Category | |
|---|---|
| <24 | 0.014390243902439025 |
| 25-29 | 0.00913312693498452 |
| 30-34 | 0.009833333333333335 |
| 35-39 | 0.02312755102040816 |
| 40-44 | 0.014549632352941176 |
| 45-49 | 0.006541125541125542 |
| 50-54 | 0.017927609427609428 |
| 55-60 | 0.0041676829268292685 |
| 60-64 | 0.0 |
| 64< | 0.0 |Ability
Ability
Ability
Age group
Age group
P<0.001
Age group
P=0.780
(J)
(K)
P=0.515
Age group
### Chart
| Category | |
|---|---|
| <24 | -0.3532317073170728 |
| 25-29 | -0.30127863777089836 |
| 30-34 | -0.311473333333334 |
| 35-39 | -0.29559693877551063 |
| 40-44 | -0.3786323529411748 |
| 45-49 | -0.3753852813852793 |
| 50-54 | -0.410553872053871 |
| 55-60 | -0.3489481707317076 |
| 60-64 | -0.3134680851063834 |
| 64< | -0.48424528301886827 |
### Chart
| Category | |
|---|---|
| <24 | 0.06705121951219521 |
| 25-29 | 0.15419195046439577 |
| 30-34 | 0.10889333333333329 |
| 35-39 | 0.15358673469387685 |
| 40-44 | 0.036159926470588355 |
| 45-49 | -0.0439740259740258 |
| 50-54 | -0.009242424242424354 |
| 55-60 | -0.04672256097560954 |
| 60-64 | -0.02203546099290777 |
| 64< | -0.1362641509433962 |Ability
Ability
Age group
P<0.001
S2 Figure
P=0.041
