# Supplementary material for: Prioritization of the Skills to Be Mastered for the Daily Jobs of Japanese Dental Hygienists
Source: Int J Dent. 2020 Jun 22;2020:4297646. doi: 10.1155/2020/4297646 (PMC7327552; doi:10.1155/2020/4297646)
Supplement: Supplementary Materials — Table S1: frequencies and item response analysis results of the seventy seven daily jobs of dental hygienists. Table S2: cross tabulations of the work-related tasks by working style and age group. Table S3: results of factor analysis of seventy seven work-related tasks. Figure S1: item response curve and item information curve for seventy-seven items. Figure S2: the mean values of ability of each cluster with respect to age groups. Figure S3: the mean values of ability of each cluster with respect to employment status (full time or part time). [file 4297646.f1.zip › 4297646.f1/Additional file 4 S3 Figuer .pptx]

## Slide 1
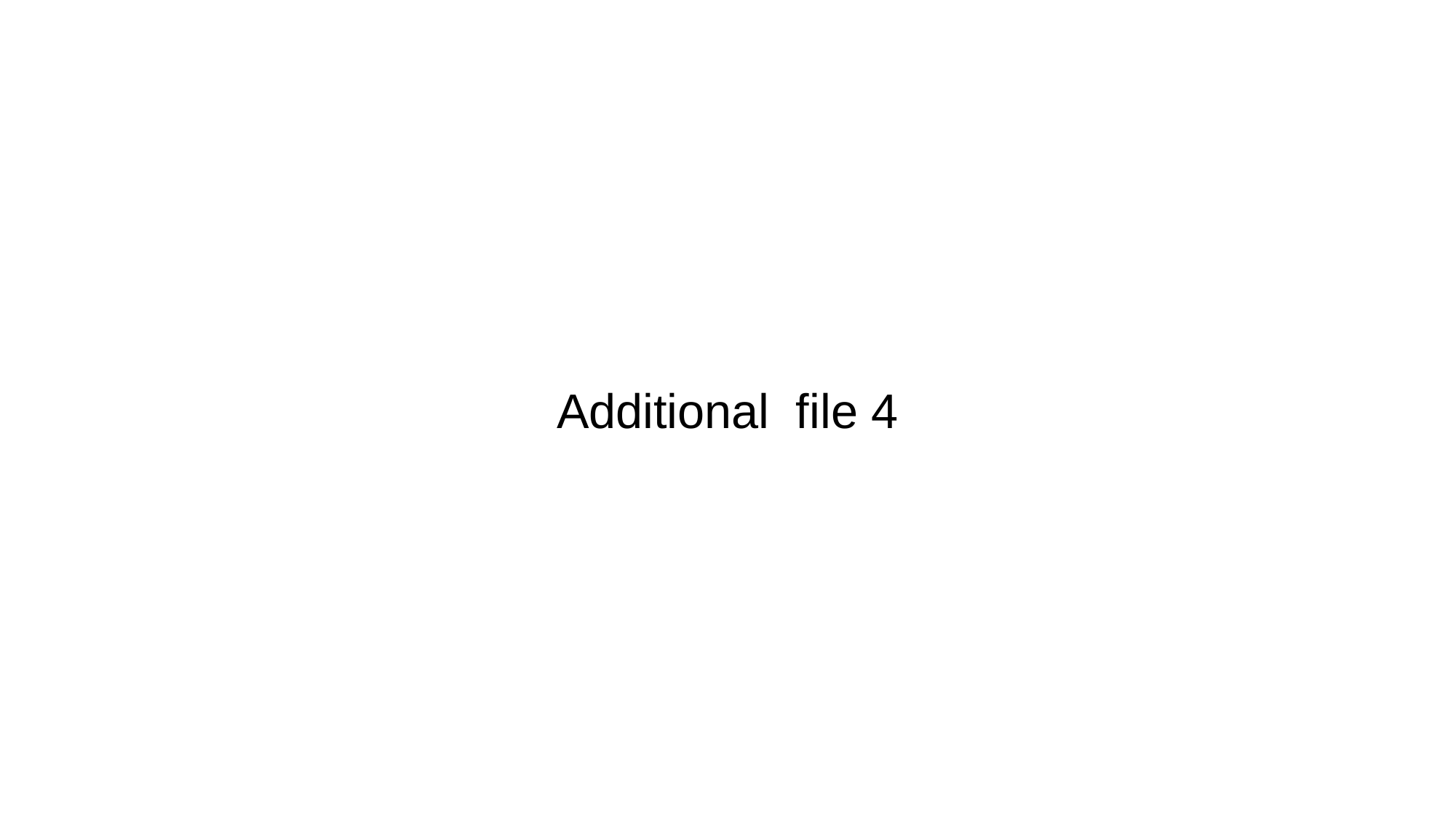

Additional file 4

## Slide 2
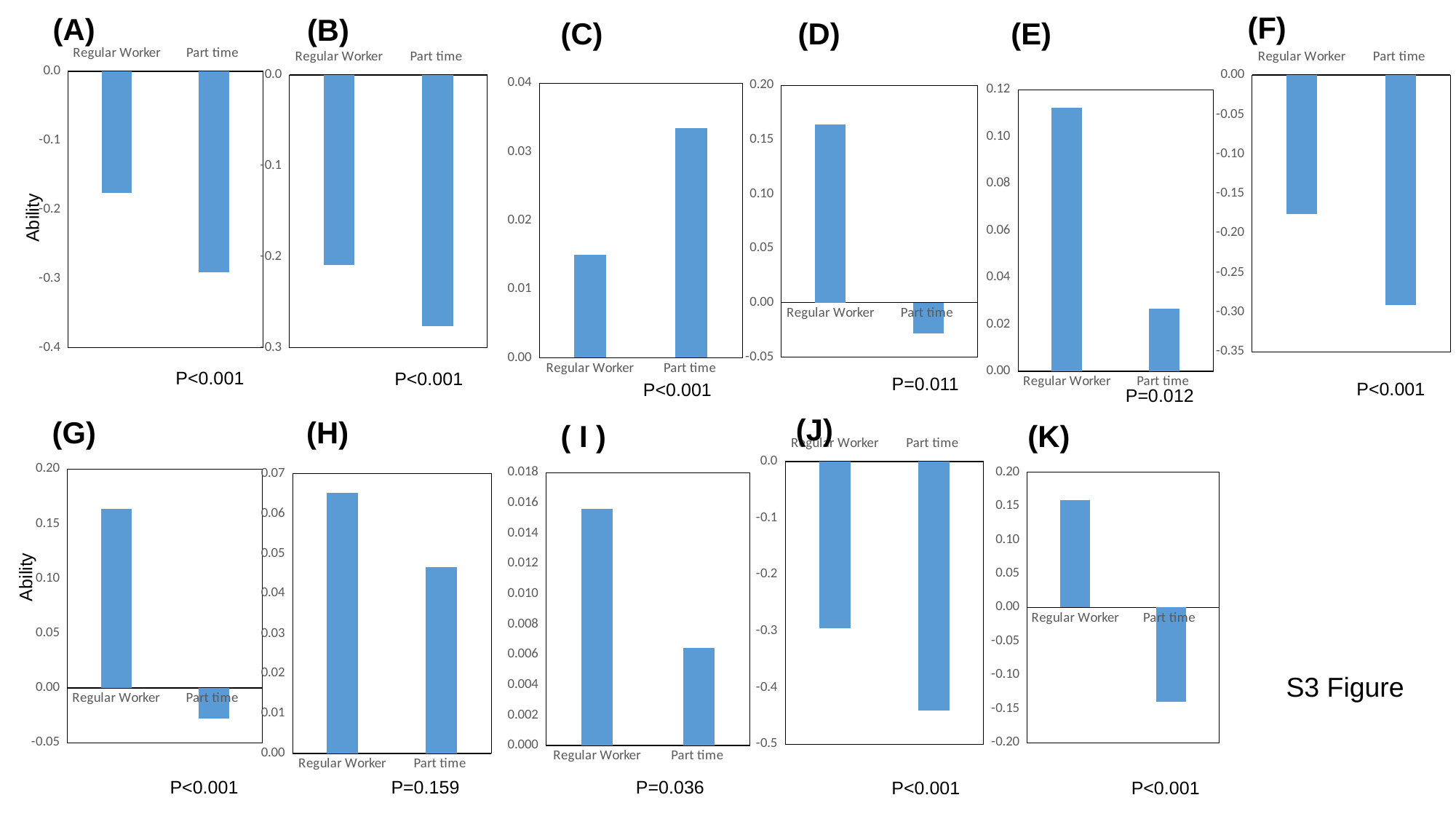

(F)
(A)
(B)
(C)
(D)
(E)
### Chart
| Category | |
|---|---|
| Regular Worker | -0.1760445382090927 |
| Part time | -0.29102410383188837 |
### Chart
| Category | |
|---|---|
| Regular Worker | -0.2093591186122848 |
| Part time | -0.2763127317676166 |
### Chart
| Category | |
|---|---|
| Regular Worker | -0.1760445382090927 |
| Part time | -0.29102410383188837 |
### Chart
| Category | |
|---|---|
| Regular Worker | 0.014997655883731836 |
| Part time | 0.03348640296662545 |
### Chart
| Category | |
|---|---|
| Regular Worker | 0.16356727613689612 |
| Part time | -0.028055624227441175 |
### Chart
| Category | |
|---|---|
| Regular Worker | 0.11219081106422776 |
| Part time | 0.026499381953028183 |Ability
P<0.001
P<0.001
P=0.011
P<0.001
P<0.001
P=0.012
(J)
(G)
(H)
(K)
( I )
### Chart
| Category | |
|---|---|
| Regular Worker | -0.2948166901078261 |
| Part time | -0.44126514215080137 |
### Chart
| Category | |
|---|---|
| Regular Worker | 0.16356727613689612 |
| Part time | -0.028055624227441175 |
### Chart
| Category | |
|---|---|
| Regular Worker | 0.015618377871542426 |
| Part time | 0.0064480840543881335 |
### Chart
| Category | |
|---|---|
| Regular Worker | 0.15871917487107295 |
| Part time | -0.13912731767614284 |
### Chart
| Category | |
|---|---|
| Regular Worker | 0.06514205344585193 |
| Part time | 0.04662608158220064 |Ability
S3 Figure
P<0.001
P=0.159
P=0.036
P<0.001
P<0.001
