# Supplementary material for: Prioritization of the Skills to Be Mastered for the Daily Jobs of Japanese Dental Hygienists
Source: Int J Dent. 2020 Jun 22;2020:4297646. doi: 10.1155/2020/4297646 (PMC7327552; doi:10.1155/2020/4297646)
Supplement: Supplementary Materials — Table S1: frequencies and item response analysis results of the seventy seven daily jobs of dental hygienists. Table S2: cross tabulations of the work-related tasks by working style and age group. Table S3: results of factor analysis of seventy seven work-related tasks. Figure S1: item response curve and item information curve for seventy-seven items. Figure S2: the mean values of ability of each cluster with respect to age groups. Figure S3: the mean values of ability of each cluster with respect to employment status (full time or part time). [file 4297646.f1.zip › 4297646.f1/Additonal file 2 S1 Figuer .pptx]

## Slide 1
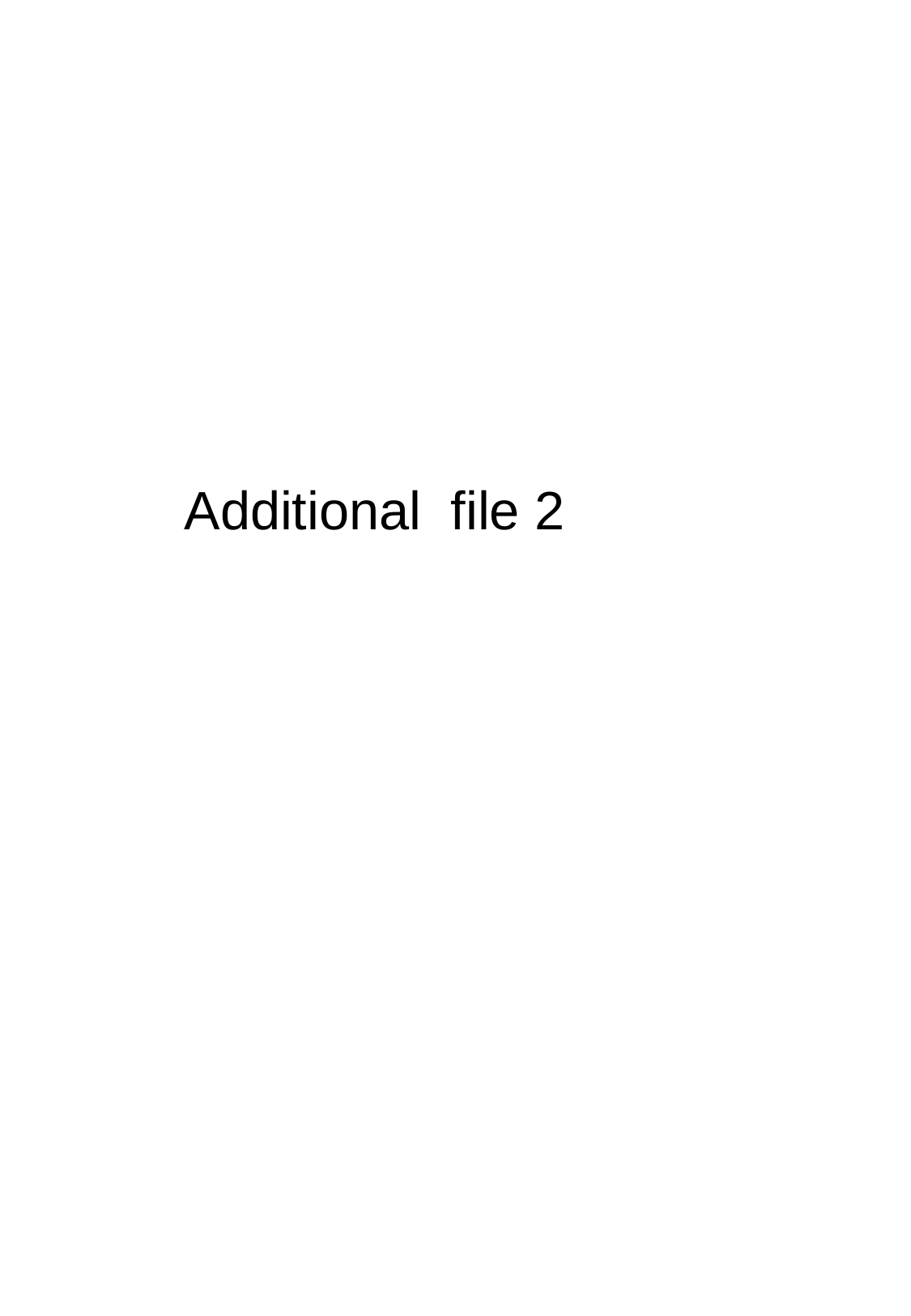

Additional file 2

## Slide 2
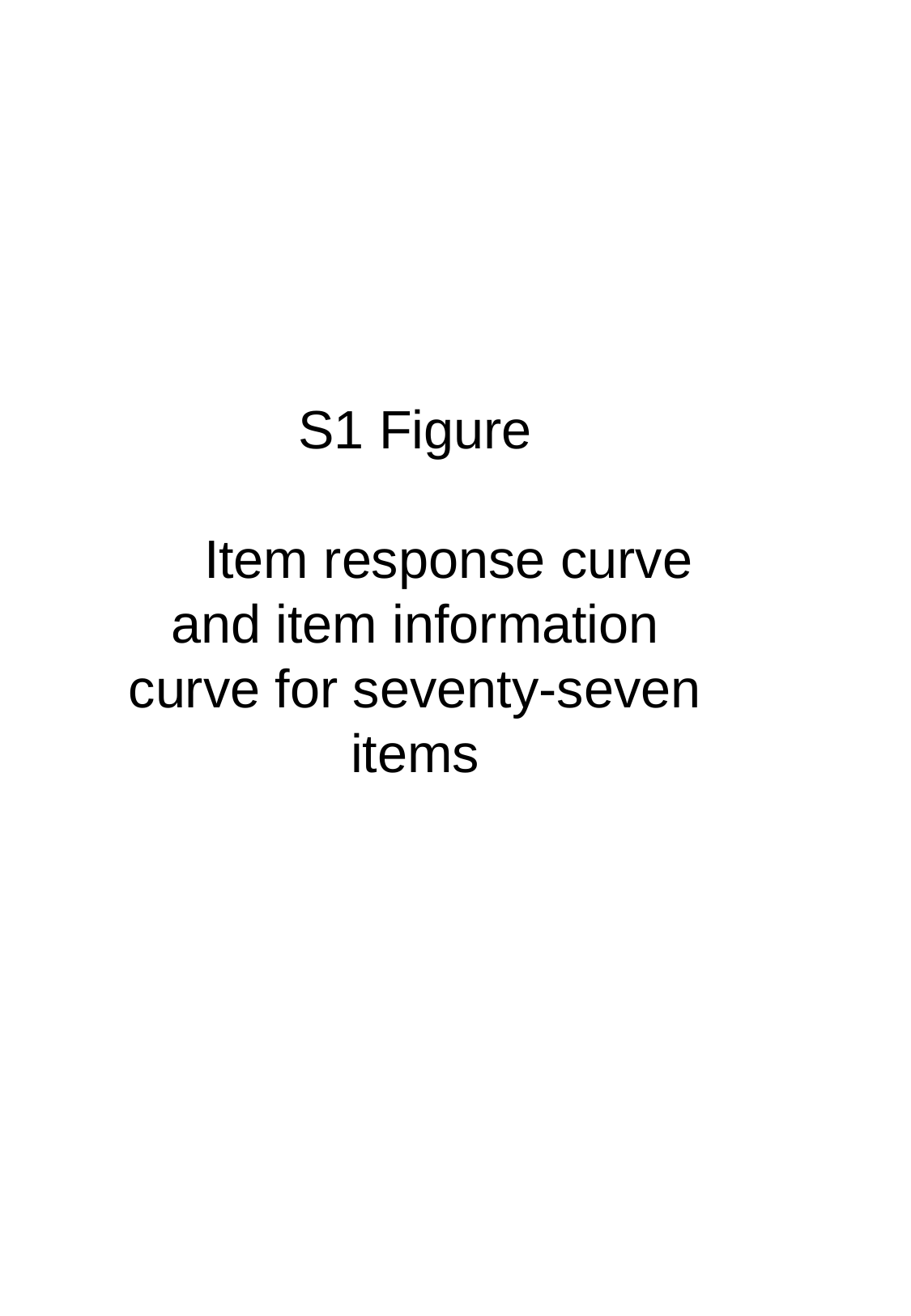

S1 Figure
　Item response curve and item information curve for seventy-seven items

## Slide 3
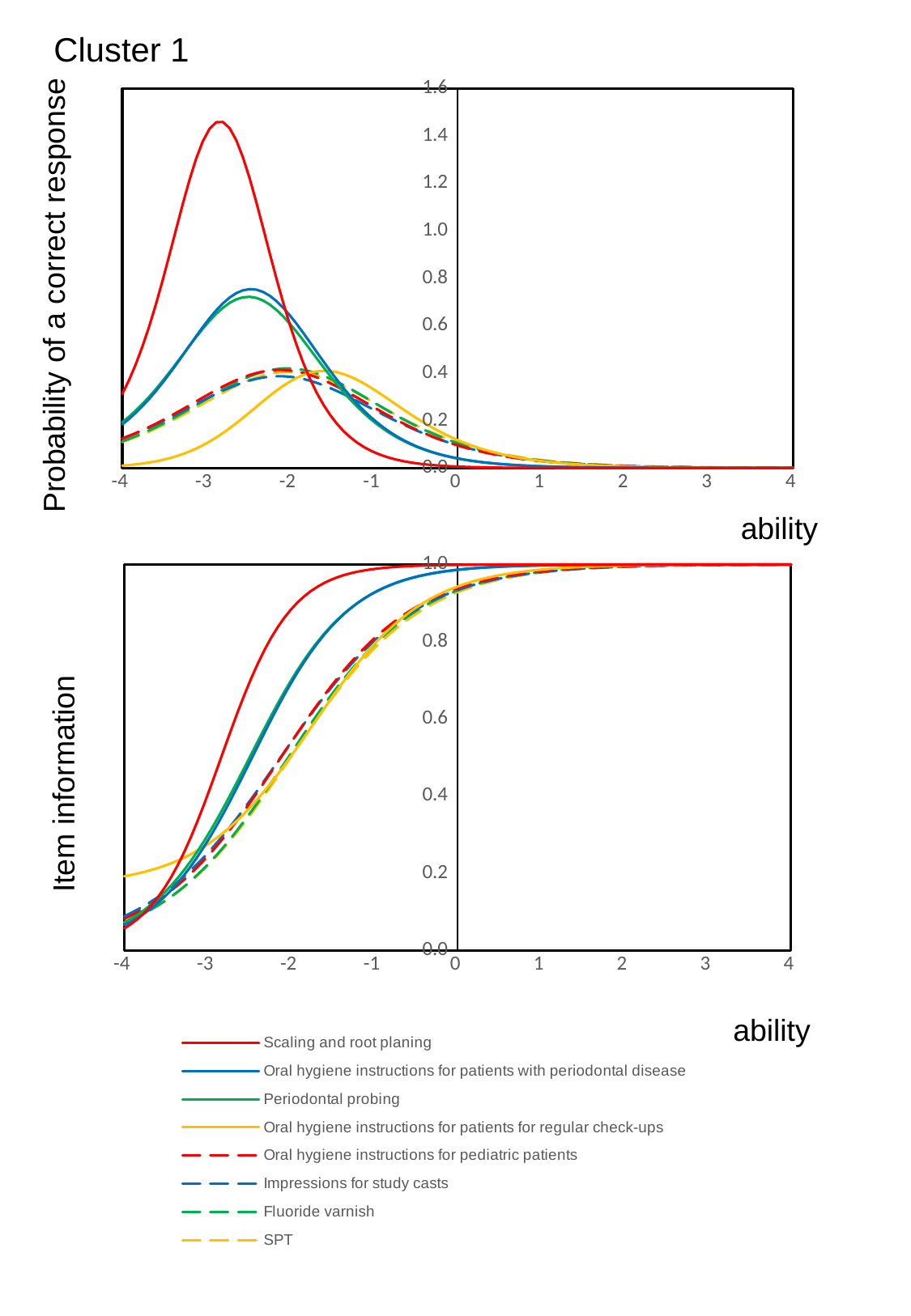

Cluster 1
### Chart
| Category | Scaling and root planing | Oral hygiene instructions for patients with periodontal disease | Periodontal probing | Oral hygiene instructions for patients for regular check-ups | Oral hygiene instructions for pediatric patients | Impressions for study casts | Fluoride varnish | SPT |
|---|---|---|---|---|---|---|---|---|Probability of a correct response
ability
### Chart
| Category | Scaling and root planing | Oral hygiene instructions for patients with periodontal disease | Periodontal probing | Oral hygiene instructions for patients for regular check-ups | Oral hygiene instructions for pediatric patients | Impressions for study casts | Fluoride varnish | SPT |
|---|---|---|---|---|---|---|---|---|Item information
ability

## Slide 4
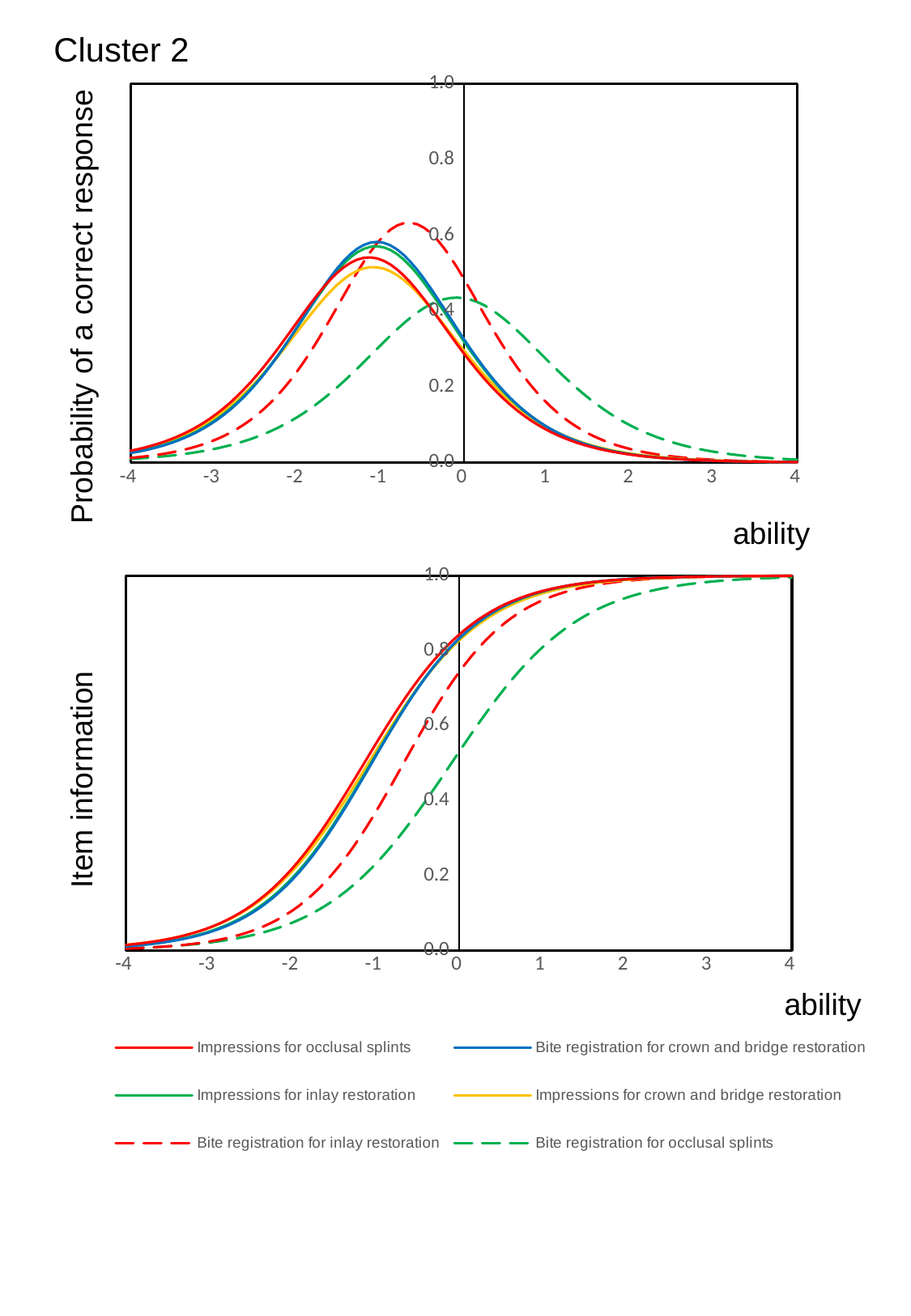

Cluster 2
### Chart
| Category | Impressions for occlusal splints | Bite registration for crown and bridge restoration | Impressions for inlay restoration | Impressions for crown and bridge restoration | Bite registration for inlay restoration | #REF! | Bite registration for occlusal splints |
|---|---|---|---|---|---|---|---|Probability of a correct response
ability
### Chart
| Category | Impressions for occlusal splints | Bite registration for crown and bridge restoration | Impressions for inlay restoration | Impressions for crown and bridge restoration | Bite registration for inlay restoration | #REF! | Bite registration for occlusal splints |
|---|---|---|---|---|---|---|---|Item information
ability

## Slide 5
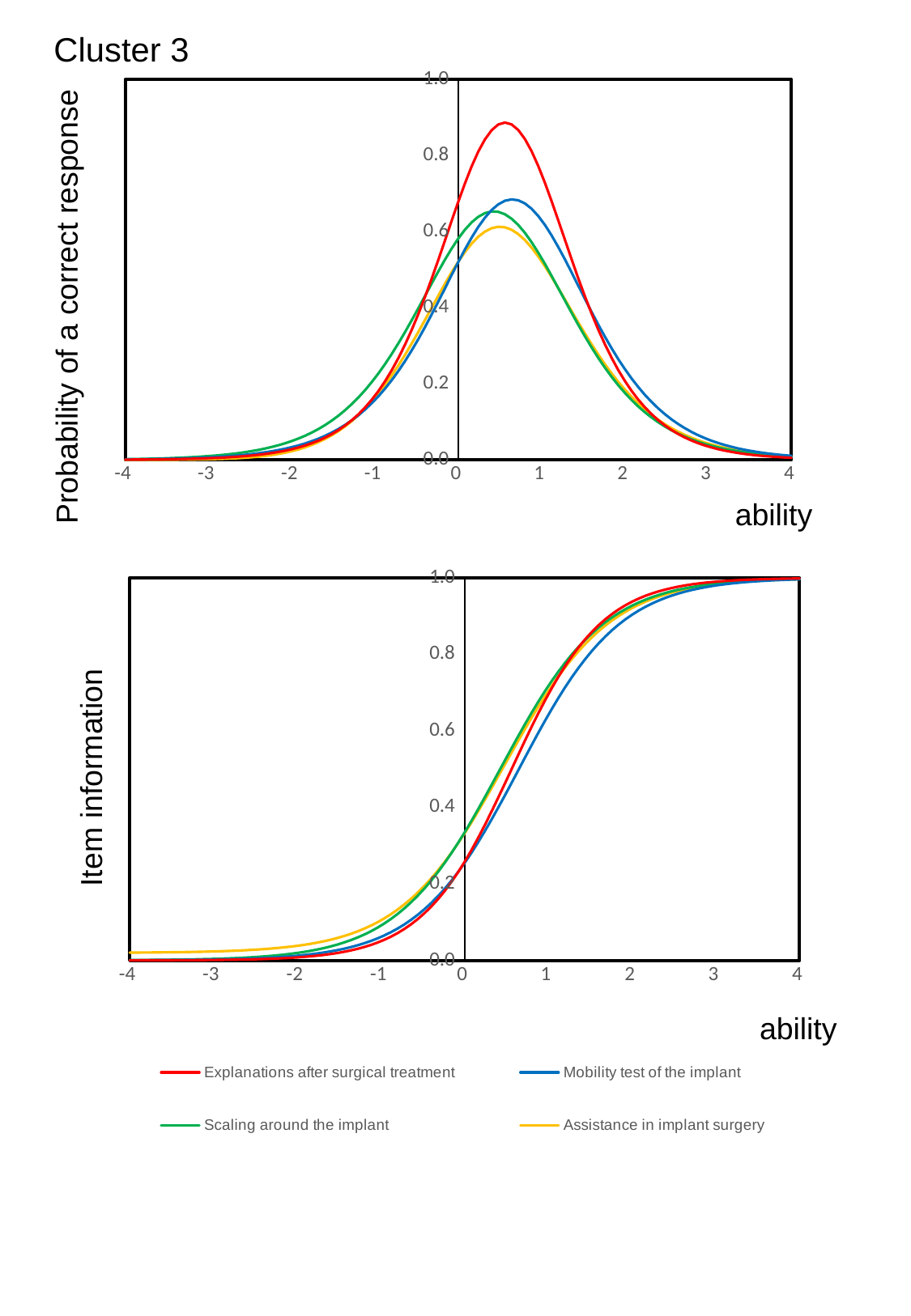

Cluster 3
### Chart
| Category | Explanations after surgical treatment | Mobility test of the implant | Scaling around the implant | Assistance in implant surgery |
|---|---|---|---|---|Probability of a correct response
ability
### Chart
| Category | Explanations after surgical treatment | Mobility test of the implant | Scaling around the implant | Assistance in implant surgery |
|---|---|---|---|---|Item information
ability

## Slide 6
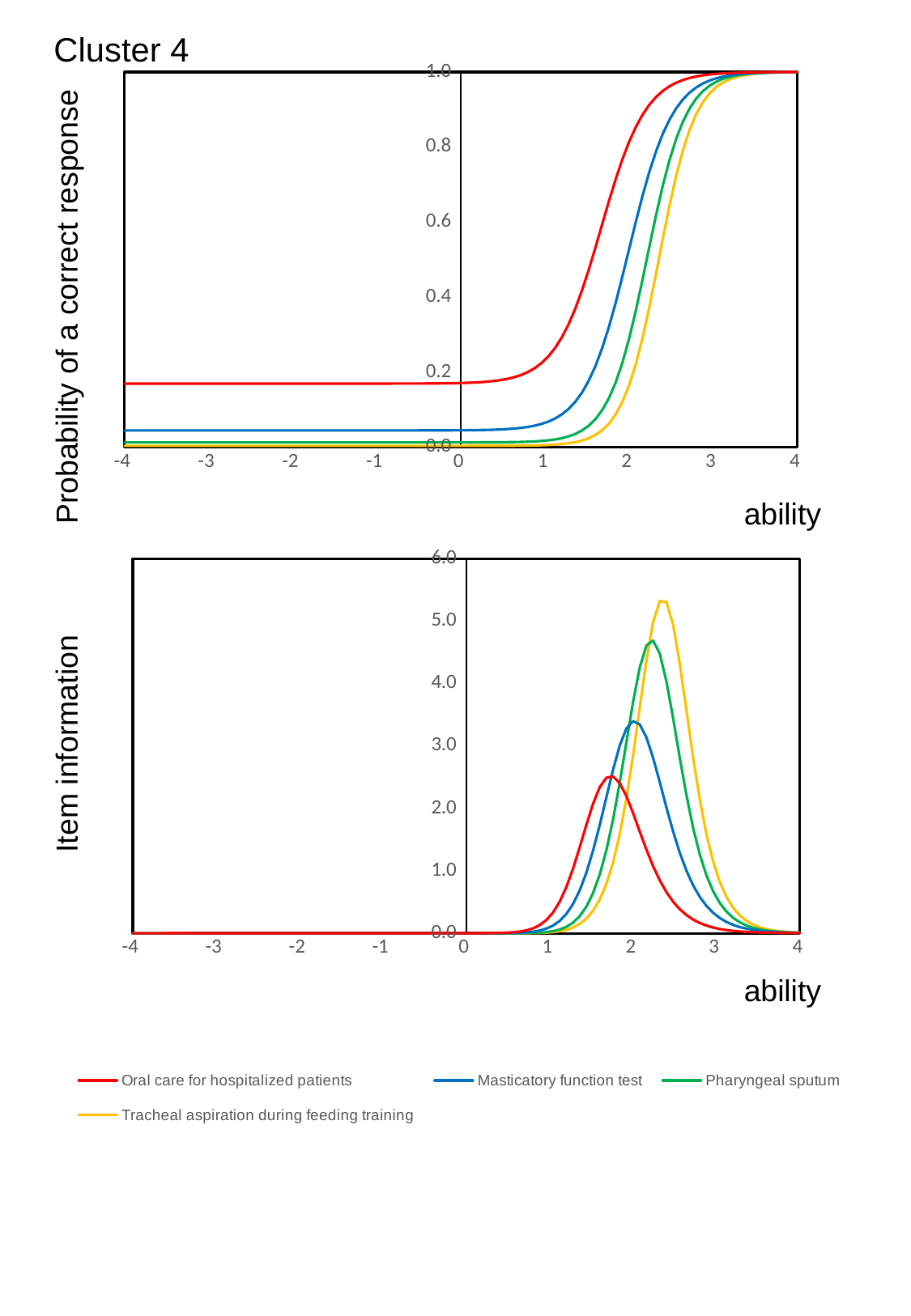

Cluster 4
### Chart
| Category | Oral care for hospitalized patients | Masticatory function test | Pharyngeal sputum | Tracheal aspiration during feeding training |
|---|---|---|---|---|Probability of a correct response
ability
### Chart
| Category | Oral care for hospitalized patients | Masticatory function test | Pharyngeal sputum | Tracheal aspiration during feeding training |
|---|---|---|---|---|Item information
ability

## Slide 7
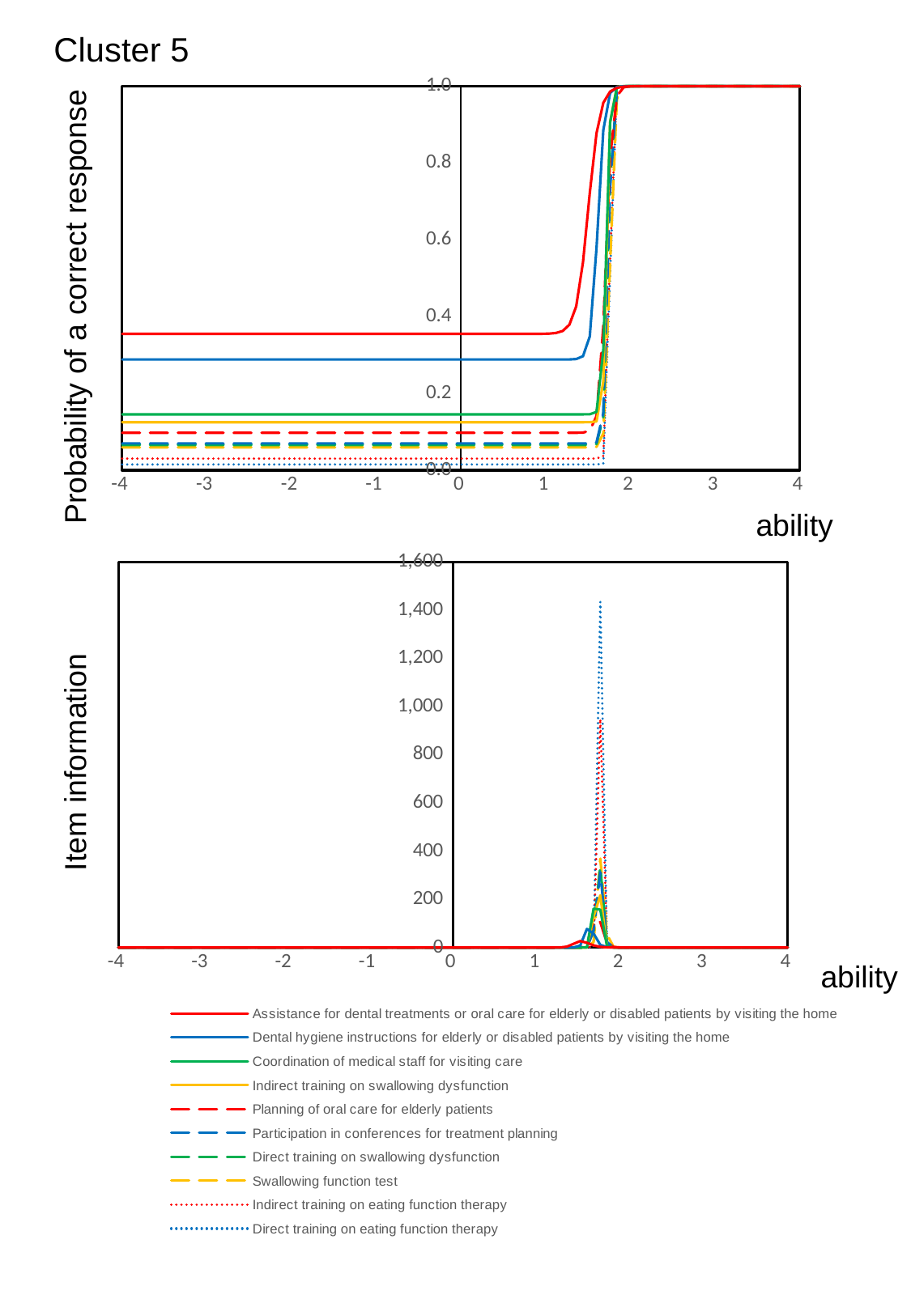

Cluster 5
### Chart
| Category | Assistance for dental treatments or oral care for elderly or disabled patients by visiting the home | Dental hygiene instructions for elderly or disabled patients by visiting the home | Coordination of medical staff for visiting care | Indirect training on swallowing dysfunction | Planning of oral care for elderly patients | Participation in conferences for treatment planning | Direct training on swallowing dysfunction | Swallowing function test | Indirect training on eating function therapy | Direct training on eating function therapy |
|---|---|---|---|---|---|---|---|---|---|---|Probability of a correct response
ability
### Chart
| Category | Assistance for dental treatments or oral care for elderly or disabled patients by visiting the home | Dental hygiene instructions for elderly or disabled patients by visiting the home | Coordination of medical staff for visiting care | Indirect training on swallowing dysfunction | Planning of oral care for elderly patients | Participation in conferences for treatment planning | Direct training on swallowing dysfunction | Swallowing function test | Indirect training on eating function therapy | Direct training on eating function therapy |
|---|---|---|---|---|---|---|---|---|---|---|Item information
ability

## Slide 8
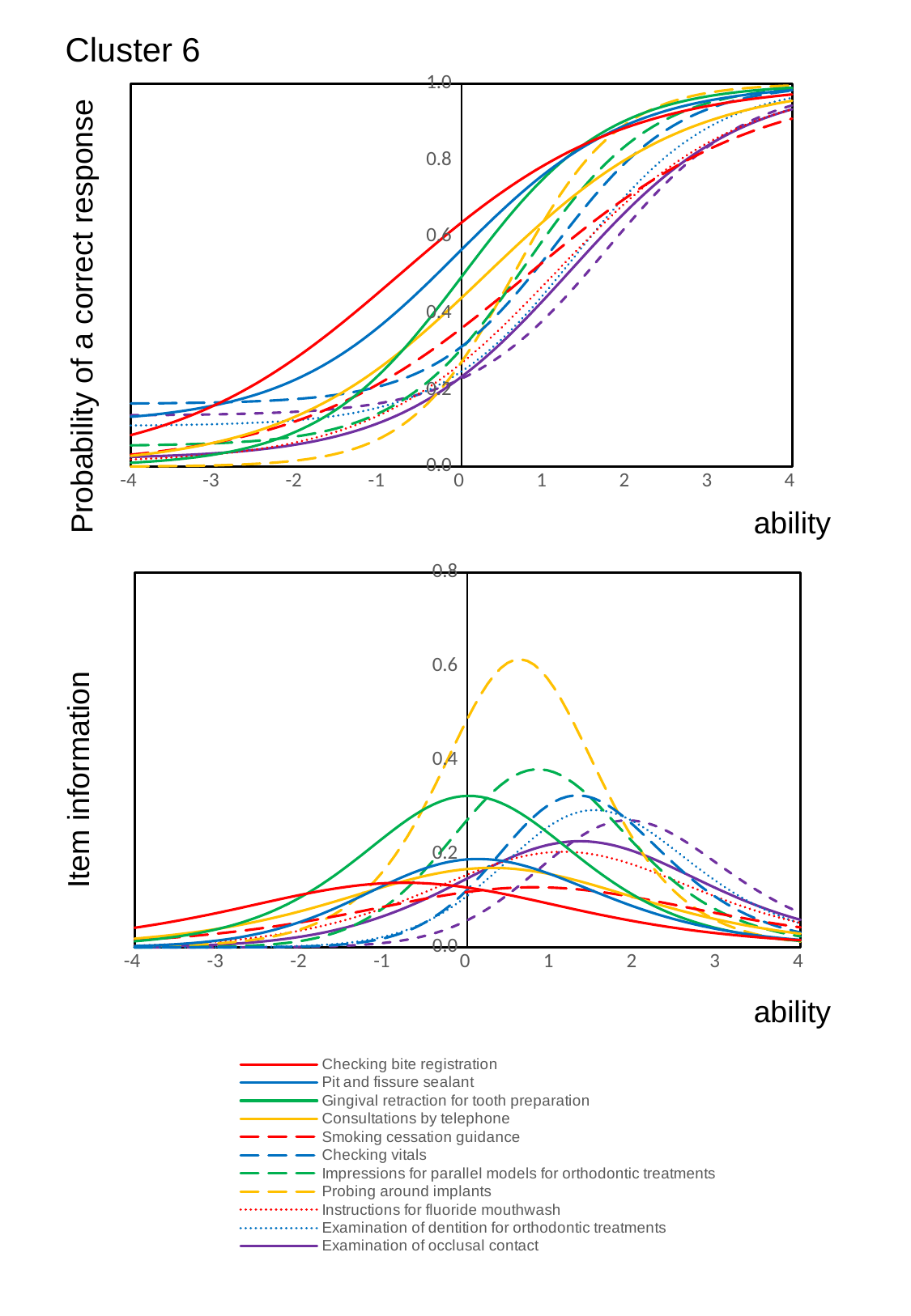

Cluster 6
### Chart
| Category | Checking bite registration | Pit and fissure sealant | Gingival retraction for tooth preparation | Consultations by telephone | Smoking cessation guidance | Checking vitals  | Impressions for parallel models for orthodontic treatments | Probing around implants | Instructions for fluoride mouthwash | Examination of dentition for orthodontic treatments | Examination of occlusal contact | Management and teaching for staff |
|---|---|---|---|---|---|---|---|---|---|---|---|---|Probability of a correct response
ability
### Chart
| Category | Checking bite registration | Pit and fissure sealant | Gingival retraction for tooth preparation | Consultations by telephone | Smoking cessation guidance | Checking vitals  | Impressions for parallel models for orthodontic treatments | Probing around implants | Instructions for fluoride mouthwash | Examination of dentition for orthodontic treatments | Examination of occlusal contact | Management and teaching for staff |
|---|---|---|---|---|---|---|---|---|---|---|---|---|Item information
ability

## Slide 9
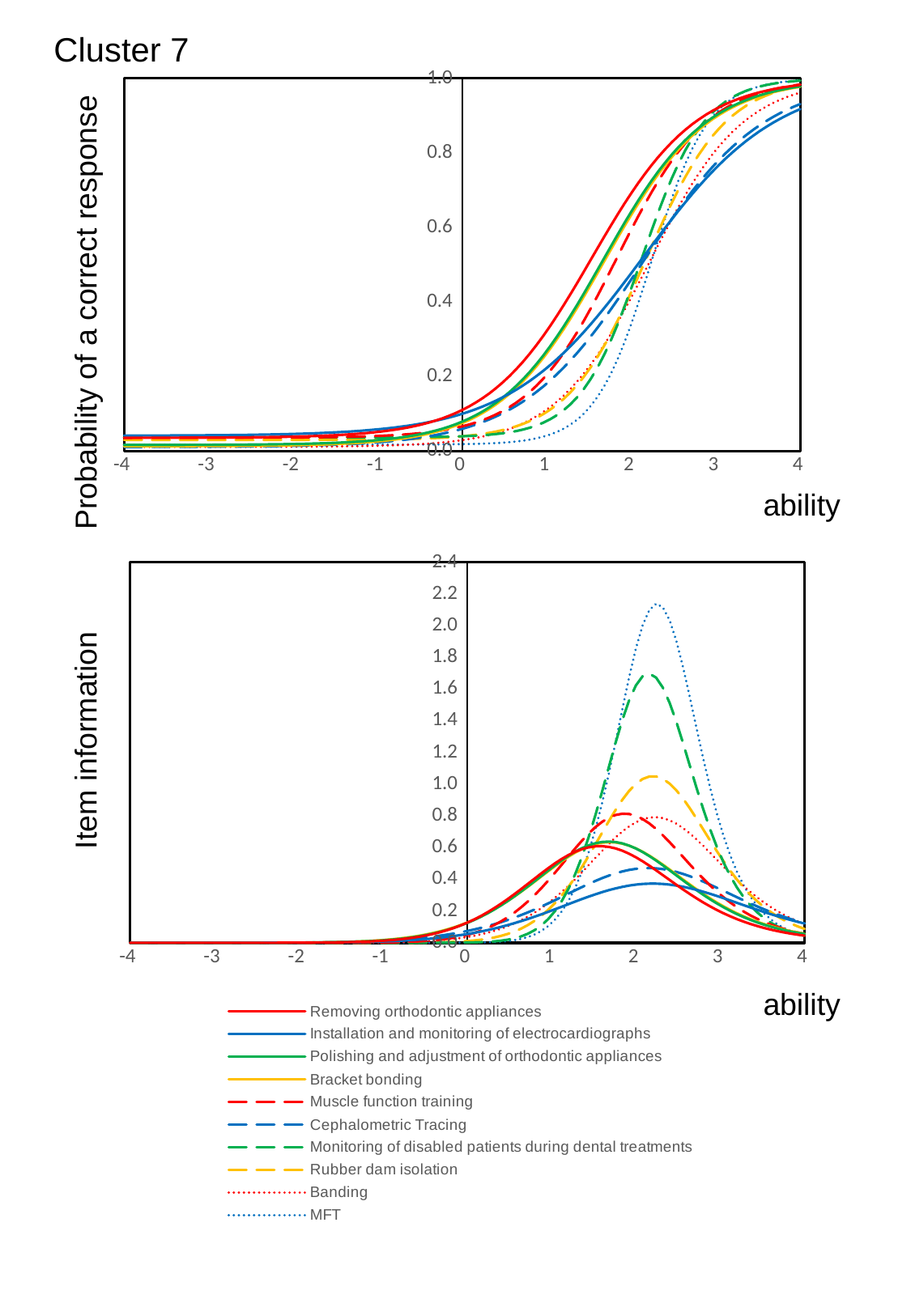

Cluster 7
### Chart
| Category | Removing orthodontic appliances | Installation and monitoring of electrocardiographs | Polishing and adjustment of orthodontic appliances | Bracket bonding | Muscle function training | Cephalometric Tracing | Monitoring of disabled patients during dental treatments | Rubber dam isolation | Banding | MFT |
|---|---|---|---|---|---|---|---|---|---|---|Probability of a correct response
ability
### Chart
| Category | Removing orthodontic appliances | Installation and monitoring of electrocardiographs | Polishing and adjustment of orthodontic appliances | Bracket bonding | Muscle function training | Cephalometric Tracing | Monitoring of disabled patients during dental treatments | Rubber dam isolation | Banding | MFT |
|---|---|---|---|---|---|---|---|---|---|---|Item information
ability

## Slide 10
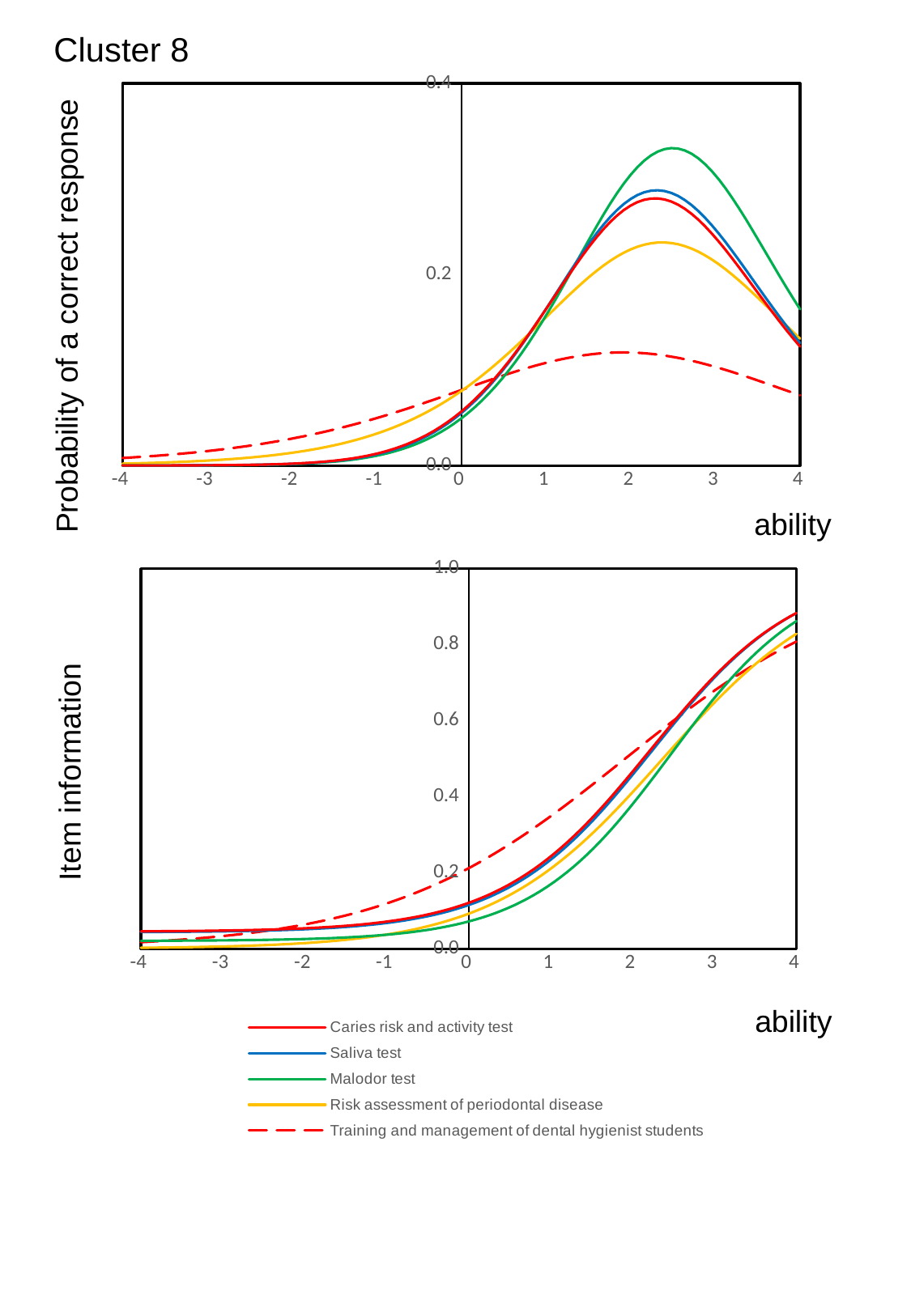

Cluster 8
### Chart
| Category | Caries risk and activity test | Saliva test | Malodor test | Risk assessment of periodontal disease | Training and management of dental hygienist students |
|---|---|---|---|---|---|Probability of a correct response
ability
### Chart
| Category | Caries risk and activity test | Saliva test | Malodor test | Risk assessment of periodontal disease | Training and management of dental hygienist students |
|---|---|---|---|---|---|Item information
ability

## Slide 11
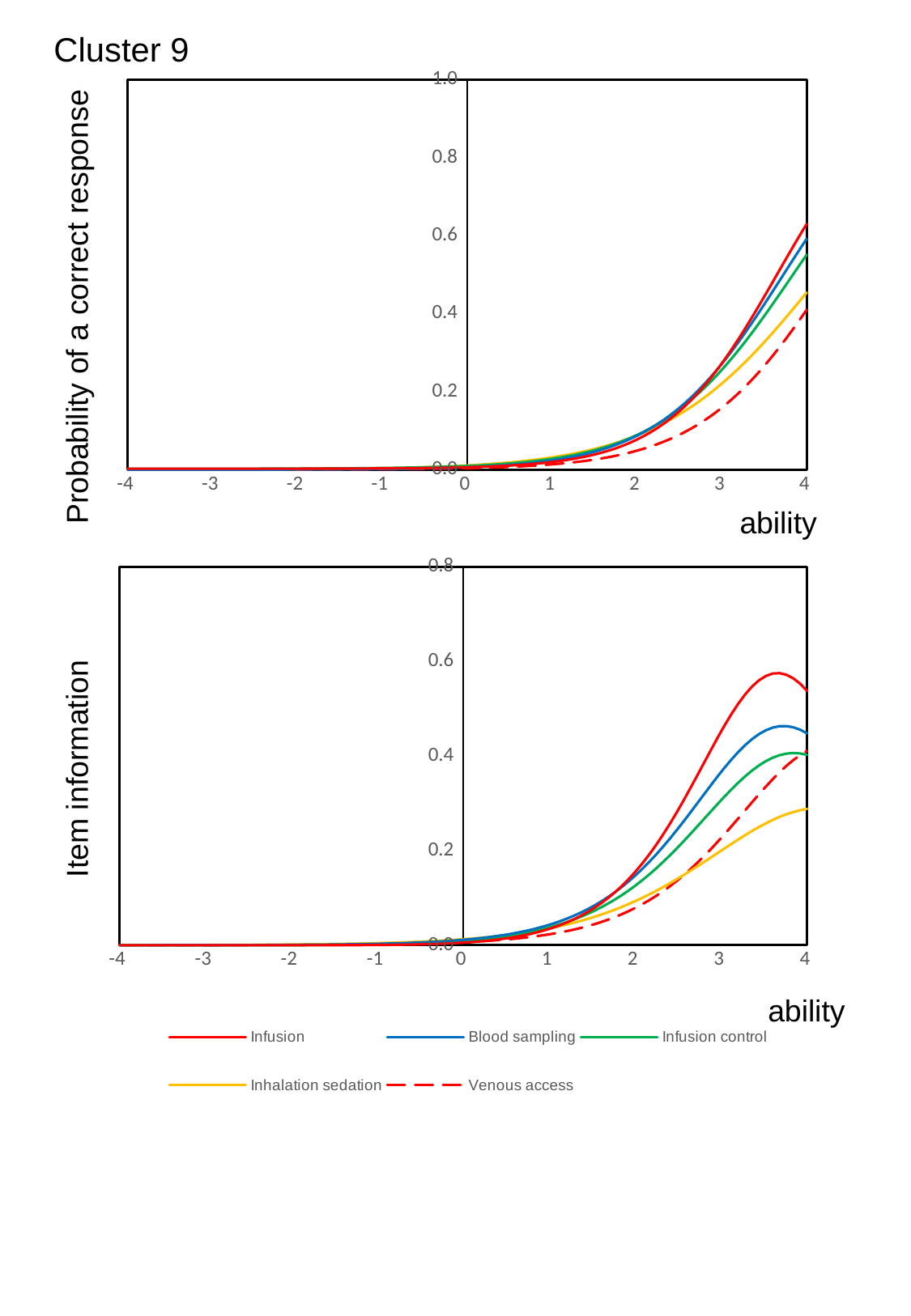

Cluster 9
### Chart
| Category | Infusion | Blood sampling | Infusion control | Inhalation sedation | Venous access |
|---|---|---|---|---|---|Probability of a correct response
ability
### Chart
| Category | Infusion | Blood sampling | Infusion control | Inhalation sedation | Venous access |
|---|---|---|---|---|---|Item information
ability

## Slide 12
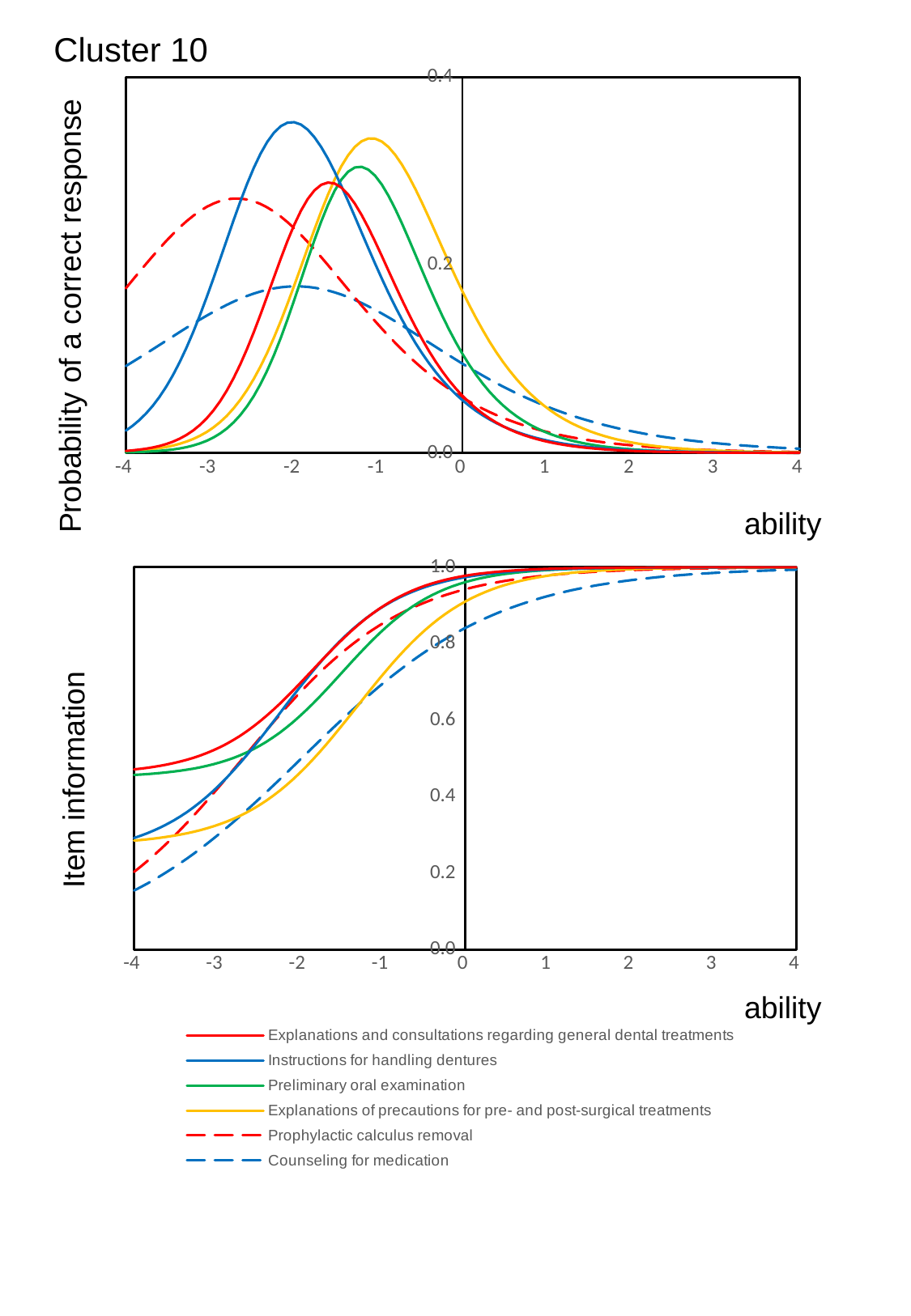

Cluster 10
### Chart
| Category | Explanations and consultations regarding general dental treatments | Instructions for handling dentures | Preliminary oral examination | Explanations of precautions for pre- and post-surgical treatments | Prophylactic calculus removal | Counseling for medication |
|---|---|---|---|---|---|---|Probability of a correct response
ability
### Chart
| Category | Explanations and consultations regarding general dental treatments | Instructions for handling dentures | Preliminary oral examination | Explanations of precautions for pre- and post-surgical treatments | Prophylactic calculus removal | Counseling for medication |
|---|---|---|---|---|---|---|Item information
ability

## Slide 13
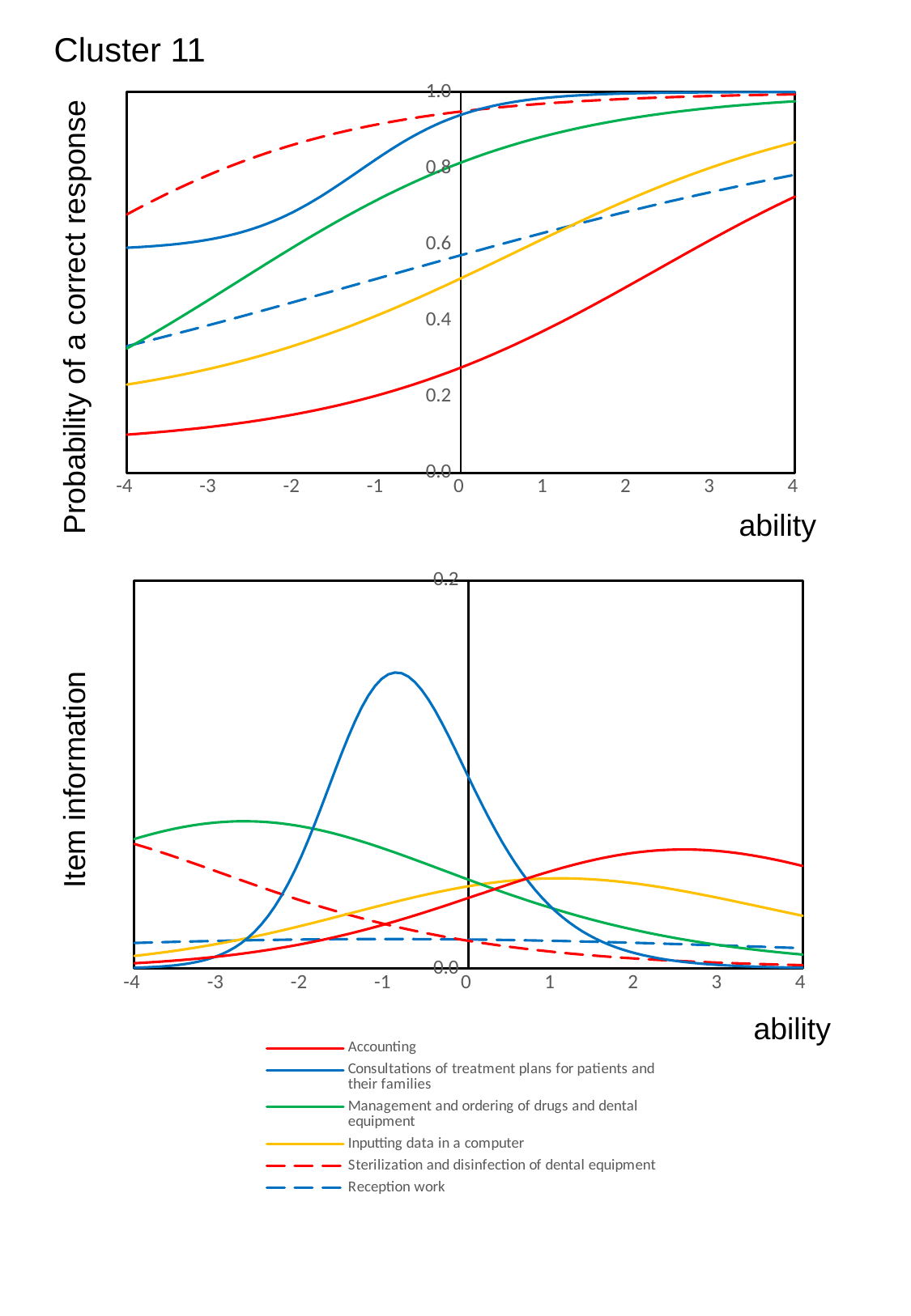

Cluster 11
### Chart
| Category | Accounting | Consultations of treatment plans for patients and their families | Management and ordering of drugs and dental equipment | Inputting data in a computer | Sterilization and disinfection of dental equipment | Reception work  |
|---|---|---|---|---|---|---|Probability of a correct response
ability
### Chart
| Category | Accounting | Consultations of treatment plans for patients and their families | Management and ordering of drugs and dental equipment | Inputting data in a computer | Sterilization and disinfection of dental equipment | Reception work  |
|---|---|---|---|---|---|---|Item information
ability
